# Cutaneous T cell lymphoma atlas reveals malignant T_H_2 cells supported by a B cell-rich tumor microenvironment

**DOI:** 10.1038/s41590-024-02018-1

**Published:** 2024-11-18

**Authors:** Ruoyan Li, Johanna Strobl, Elizabeth F. M. Poyner, Aya Balbaa, Fereshteh Torabi, Pavel V. Mazin, Nana-Jane Chipampe, Emily Stephenson, Ciro Ramírez-Suástegi, Vijaya Baskar Mahalingam Shanmugiah, Louis Gardner, Bayanne Olabi, Rowen Coulthard, Rachel A. Botting, Nina Zila, Elena Prigmore, Nusayhah H. Gopee, Marta A. Chroscik, Efpraxia Kritikaki, Justin Engelbert, Issac Goh, Hon Man Chan, Harriet F. Johnson, Jasmine Ellis, Victoria Rowe, Win Tun, Gary Reynolds, Dexin Yang, April Rose Foster, Laure Gambardella, Elena Winheim, Chloe Admane, Benjamin Rumney, Lloyd Steele, Laura Jardine, Julia Nenonen, Keir Pickard, Jennifer Lumley, Philip Hampton, Simeng Hu, Fengjie Liu, Xiangjun Liu, David Horsfall, Daniela Basurto-Lozada, Louise Grimble, Chris M. Bacon, Sophie C. Weatherhead, Hanna Brauner, Yang Wang, Fan Bai, Nick J. Reynolds, Judith E. Allen, Constanze Jonak, Patrick M. Brunner, Sarah A. Teichmann, Muzlifah Haniffa

**Affiliations:** 1 Wellcome Sanger Institute, Wellcome Genome Campus, Hinxton, UK; 2 Department of Systems Biology, The University of Texas MD Anderson Cancer Center, Houston, TX USA; 3 MD Anderson UTHealth Graduate School of Biomedical Sciences, Houston, TX USA; 4 Department of Dermatology, Medical University of Vienna, Vienna, Austria; 5 Biosciences Institute, Newcastle University, Newcastle, UK; 6 Department of Dermatology and NIHR Newcastle Biomedical Research Centre, Newcastle, Hospitals NHS Foundation Trust, Newcastle upon Tyne, UK; 7 NovoPath, Department of Cellular Pathology, Newcastle Hospitals NHS Foundation Trust, Newcastle upon Tyne, UK; 8 Section Biomedical Science, University of Applied Sciences FH Campus Wien, Vienna, Austria; 9 Center for Immunology and Inflammatory Diseases, Massachusetts General Hospital, Boston, MA USA; 10 Division of Dermatology, Department of Medicine, Solna and Center for Molecular Medicine, Karolinska Institutet, Stockholm, Sweden; 11 Translational and Clinical Research Institute, Newcastle University, Newcastle upon Tyne, UK; 12 Biomedical Pioneering Innovation Center and School of Life Sciences, Peking University, Beijing, China; 13 Department of Dermatology and Venerology, Peking University First Hospital, Beijing, China; 14 Wolfson Childhood Cancer Research Centre, Translational and Clinical Research Institute, Newcastle University, Newcastle upon Tyne, UK; 15 Department of Cellular Pathology, Newcastle upon Tyne Hospitals NHS Foundation Trust, Newcastle upon Tyne, UK; 16 Department of Dermatology, Karolinska University Hospital, Stockholm, Sweden; 17 Lydia Becker Institute of Immunology and Inflammation, School of Biological Sciences, Faculty of Biology, Medicine and Health, Manchester Academic Health Science Centre, University of Manchester, Manchester, UK; 18 Department of Dermatology, Icahn School of Medicine at Mount Sinai, New York, NY USA; 19 Cambridge Stem Cell Institute, Jeffrey Cheah Biomedical Centre, Cambridge Biomedical Campus, University of Cambridge, Cambridge, UK; 20 Department of Medicine, University of Cambridge, Cambridge, UK

**Keywords:** Tumour immunology, Tumour immunology, Sequencing

## Abstract

Cutaneous T cell lymphoma (CTCL) is a potentially fatal clonal malignancy of T cells primarily affecting the skin. The most common form of CTCL, mycosis fungoides, can be difficult to diagnose, resulting in treatment delay. We performed single-cell and spatial transcriptomics analysis of skin from patients with mycosis fungoides-type CTCL and an integrated comparative analysis with human skin cell atlas datasets from healthy and inflamed skin. We revealed the co-optation of T helper 2 (T_H_2) cell-immune gene programs by malignant CTCL cells and modeling of the tumor microenvironment to support their survival. We identified MHC-II^+^ fibroblasts and dendritic cells that can maintain T_H_2 cell-like tumor cells. CTCL tumor cells are spatially associated with B cells, forming tertiary lymphoid structure-like aggregates. Finally, we validated the enrichment of B cells in CTCL and its association with disease progression across three independent patient cohorts. Our findings provide diagnostic aids, potential biomarkers for disease staging and therapeutic strategies for CTCL.

## Abstract (web summary)

Haniffa and colleagues provide diagnostic aids, potential biomarkers for disease staging and therapeutic strategies for cutaneous T cell lymphoma.

## Main

Cutaneous T cell lymphoma (CTCL) is a rare disease^1^ and a subgroup of non-Hodgkin’s lymphoma, with mycosis fungoides being the most common subtype, with an incidence of 5.42 per million in the USA^2^. Early stage mycosis fungoides (stages I–IIA^3^) typically presents in the skin with patches and plaques, which can be mistaken for benign inflammatory conditions such as atopic dermatitis (AD) and psoriasis, posing a challenge for clinical and histological diagnosis^4,5^. Although indolent in most, one-third of patients with early stage mycosis fungoides can progress to advanced-stage disease (≥IIB), with a low overall survival rate^6,7^. Malignant T cells in advanced-stage mycosis fungoides are typically central memory-like CD4^+^ clones characterized by high inter-donor variability^8^ and high tumor mutational burden^9^, but very little is known about their molecular characteristics.

Identification of reliable diagnostic hallmarks for CTCL across patients has been limited by its nonspecific histopathological features, as well as the heterogeneity and proposed plasticity of malignant T cells^10^. Current diagnosis is mainly based on correlation of clinical and nonspecific histopathological features, including T cell epidermotropism, band-like dermal infiltrates and fibrosis of the papillary dermis^11^, all of which can also be observed in benign inflammatory skin conditions. As such, early CTCL has been termed ‘the great imitator’^12^. Research into CTCL has traditionally focused on tumor cells in peripheral blood from patients with advanced disease^13,14^. More recent studies on skin lesions from patients with CTCL have largely been on small patient numbers^15^ and primarily focused on tumor cells^16^. Molecular characterization of malignant T cells has led to noncurative treatment options for advanced CTCL, including a monoclonal antibody directed against CCR4 (mogamulizumab)^17^ and a CD30 antibody–drug conjugate (brentuximab vedotin)^18^. A subset of patients has also been shown to respond to anti-PD-1 (anti-programmed death cell protein 1) immunotherapy^19,20^.

In the present study, we aimed to achieve a holistic understanding of tumor cells and the tumor microenvironment (TME) in lesional skin from mycosis fungoides-type CTCL (referred to as CTCL hereafter). We performed single-cell RNA sequencing (scRNA-seq), T cell receptor (TCR) sequencing and integrated data from previous studies^9,16,21^ to derive by far the largest scRNA-seq skin cell atlas of CTCL from 45 patients. We performed spatial transcriptomics on skin biopsies from patients with early and advanced-stage CTCL to validate our findings in situ and compared our findings with scRNA-seq and bulk RNA-seq datasets of healthy skin, AD and psoriasis^22–24^ to identify CTCL-specific cellular and molecular features. Our study identified a predominance of T helper 2 (T_H_2) cell-like malignant T cells in CTCL tumors, which were probably supported by major histocompatibility complex class II-positive (MHC-II^+^) fibroblasts and dendritic cells (DCs) within the TME. In addition, we demonstrated an association of malignant T_H_2 cell-like cells with B cell aggregations in samples from patients with progressive disease. Finally, we demonstrated the presence of tertiary lymphoid structures (TLSs) in CTCL lesional skin. These findings reflect new vantage points for CTCL diagnosis and treatment.

## Results

### Cellular composition of CTCL tumors and microenvironment

**Fig. 1:**
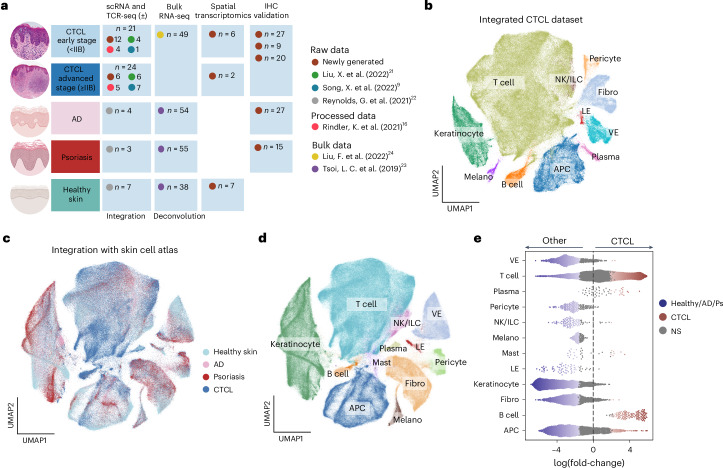
Overview of the CTCL dataset and comparisons to skin cell atlas. **a**, Detailed summary of newly generated data and integrated external datasets^9,16,21–24^ in the present study. **b**, Overall UMAP showing major cell types in our CTCL dataset. **c**, UMAPs showing the integrated CTCL and skin cell atlas data colored by diseases. **d**, UMAPs showing the broad cell types in the CTCL and skin cell atlas integrated object. **e**, Beeswarm plot of the log-transformed fold-changes in abundance of cells in CTCL versus those in healthy skin, AD and psoriasis from the skin cell atlas. Differential abundance neighborhoods at a false discovery rate (FDR) of 10% are colored. APC, antigen-presenting cells; Fibro, fibroblasts; ILC, innate lymphoid cells; LE, lymphatic endothelial cells; Melano, melanocytes; NS, not significant; VE, vascular endothelial cells.

**Extended Data Fig. 1:**
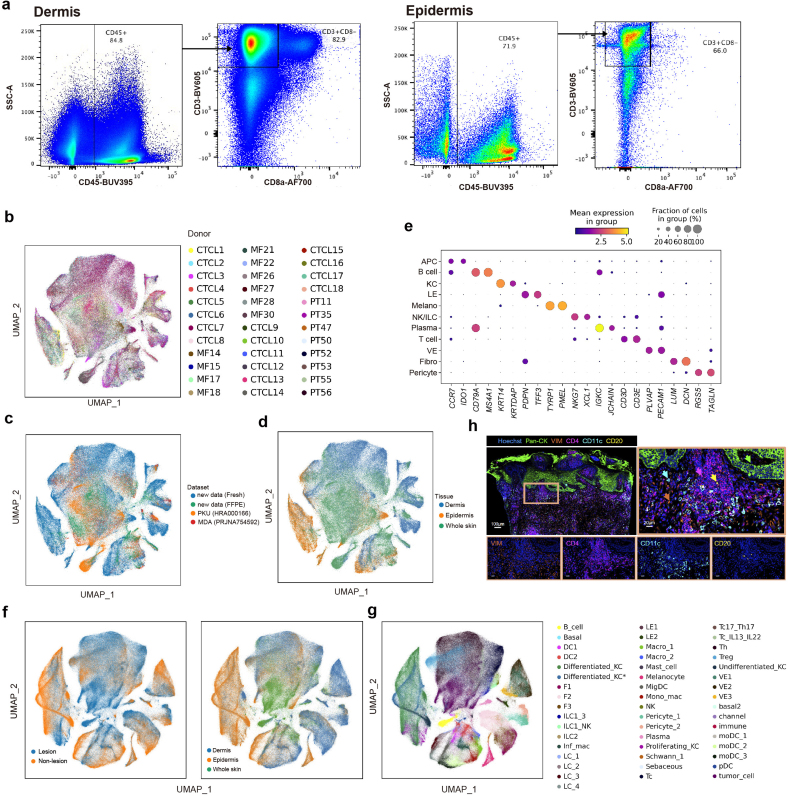
Overview of the data and data integration. **a**, Representative FACS gating strategy for scRNA-seq for dermal (top) and epidermal (bottom) samples. Plots follow on from classical live singlet gating using DAPI and FSC-A/FSC-H/SSC-W respectively. **b**, UMAP visualisation of the current CTCL data object coloured by patients. **c** and **d**, UMAPs of the current CTCL dataset coloured by datasets (**c**) and skin tissues (**d**). **e**, Gene expression dot plot of marker genes for broad cell types. Dot colour indicates log-transformed and normalised expression value. Dot size indicates the percentage of cells in each cell type expressing a given gene. **f**, UMAPs showing the integrated CTCL and skin cell atlas data coloured by lesion/non-lesion and skin tissues. **g**, UMAP showing the fine-grained annotations of cell types in the integrated CTCL and skin cell atlas dataset. Labels (annotations) are transferred from the skin cell atlas using CellTypist^25,26^ except for ‘tumour cell’ which is inferred based on CNVs. **h**, A multiplex protein immunofluorescence image (Rarecyte) showing a representative view of CTCL TME. Scale bars: 100 μm (zoomed-out) and 20 μm (zoomed-in). Image representative of n = 8 patients.

We generated scRNA-seq data (10x Chromium platform) from 18 patients with CTCL. For eight patients, we separated epidermis and dermis before scRNA-seq profiling (including TCR enrichment) all live cells from the CD45^+^CD8^+^, CD45^+^CD8^−^ and CD45^−^ fractions using FACS isolation (Extended Data Fig. 1a and Supplementary Table 1). For ten patients, we generated probe-based scRNA-seq (10× Flex) data from archival formalin-fixed paraffin-embedded (FFPE) CTCL skin samples. In addition, we integrated data from three published CTCL scRNA-seq studies^9,16,21^ to generate a CTCL skin cell atlas (45 patients in total: 21 early disease and 24 advanced disease) (Fig. 1a and Supplementary Table 1). For scRNA-seq datasets with available raw data (both newly generated and published: 36 out of 45 patients), we used a consistent analysis pipeline to process the data and integrate them (Methods). After ambient RNA removal, doublet removal and quality control, we captured approximately 420,000 single cells that could be broadly categorized into 11 cell types based on the expression of canonical marker genes (Fig. 1b and Extended Data Fig. 1b–e).

We integrated our scRNA-seq CTCL data with an existing human skin cell atlas dataset^22^, which includes healthy, AD and psoriasis skin to distinguish CTCL-specific features from common inflammatory skin disorders (Fig. 1c,d and Extended Data Fig. 1f). We performed label transfer from the skin cell atlas using CellTypist^25,26^ to annotate cell states in the integrated dataset (Extended Data Fig. 1g). Differential abundance testing using Milo^27^ was performed to interrogate differences in cellular abundance for the broad cell types seen in CTCL compared with healthy skin, AD and psoriasis (Fig. 1e). We observed an expected enrichment of T cells but also enrichment of B cells, plasma cells and mast cells in CTCL (Fig. 1e). The enrichment of B cells is surprising because they are not usually present in the skin, whether in healthy, AD or psoriasis contexts^22^.

**Fig. 2:**
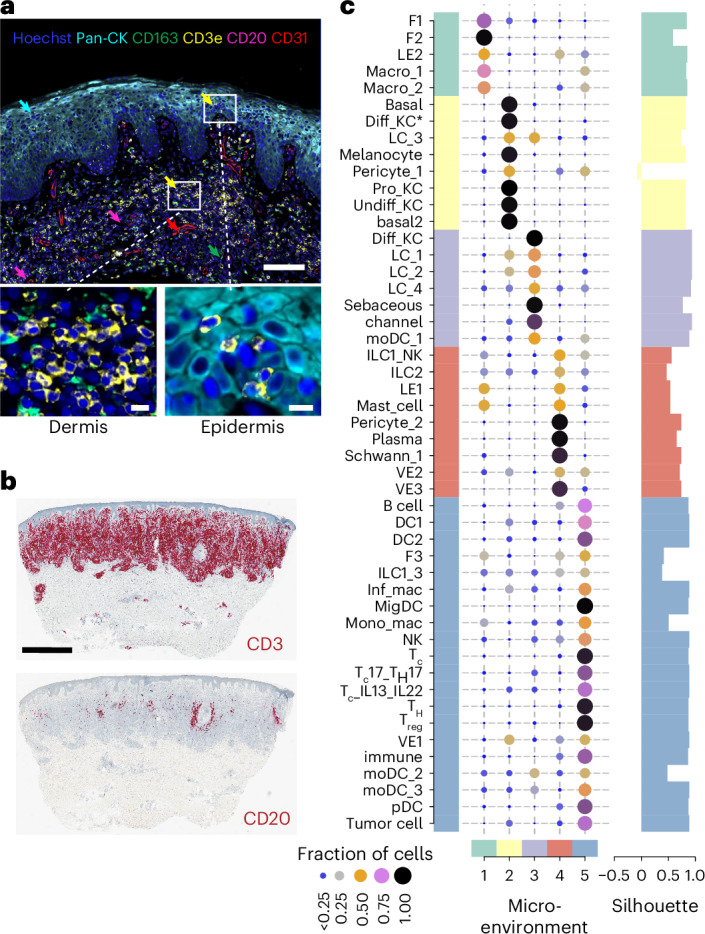
Spatial characterization of CTCL TME. **a**, A multiplex protein immunofluorescence image (RareCyte) showing a representative view of CTCL TME. Scale bars, 100 μm and 10 μm (zoomed in). **b**, IHC staining for CD3 and CD20 in a representative sample. Scale bar, 1 mm. **c**, Left, dot plot showing estimated NMF weights of cell types across NMF factors (NMF-based microenvironment) using cell2location^28^. The fractions of cells across factors are shown. Right, silhouette score showing the similarity of a cell type to its own microenvironment compared with other microenvironments. Higher values indicate better clustering.

For spatial validation, we performed spatial transcriptomics on 23 skin tissue sections (8 CTCL and 15 healthy skin) from 15 donors (10x Visium) and multiplex protein immunofluorescence analysis (RareCyte). A representative view of CTCL TME using multi-color immunofluorescence imaging showed T cells in both epidermis and dermis, together with myeloid cells and B cells surrounding dermal blood vessels (Fig. 2a and Extended Data Fig. 1h). We further confirmed the presence of B cells in CTCL by immunohistochemical (IHC) staining (Fig. 2b). We mapped the fine-grained annotated cell types to their spatial locations in skin tissues using cell2location^28^ and performed non-negative matrix factorization (NMF) of estimated cell-type abundance to identify the spatial co-occurrence of cell types. Analysis revealed malignant T cells sharing an NMF-based microenvironment (NMF factor) with fibroblasts, DCs and B cells (microenvironment 5) as well as undifferentiated and differentiated keratinocytes (microenvironment 2) (Fig. 2c). Our CTCL scRNA-seq and spatial transcriptomics data resource can be explored using WebAtlas^29^ (https://collections.cellatlas.io/ctcl).

### Malignant T cells in CTCL

**Extended Data Fig. 2:**
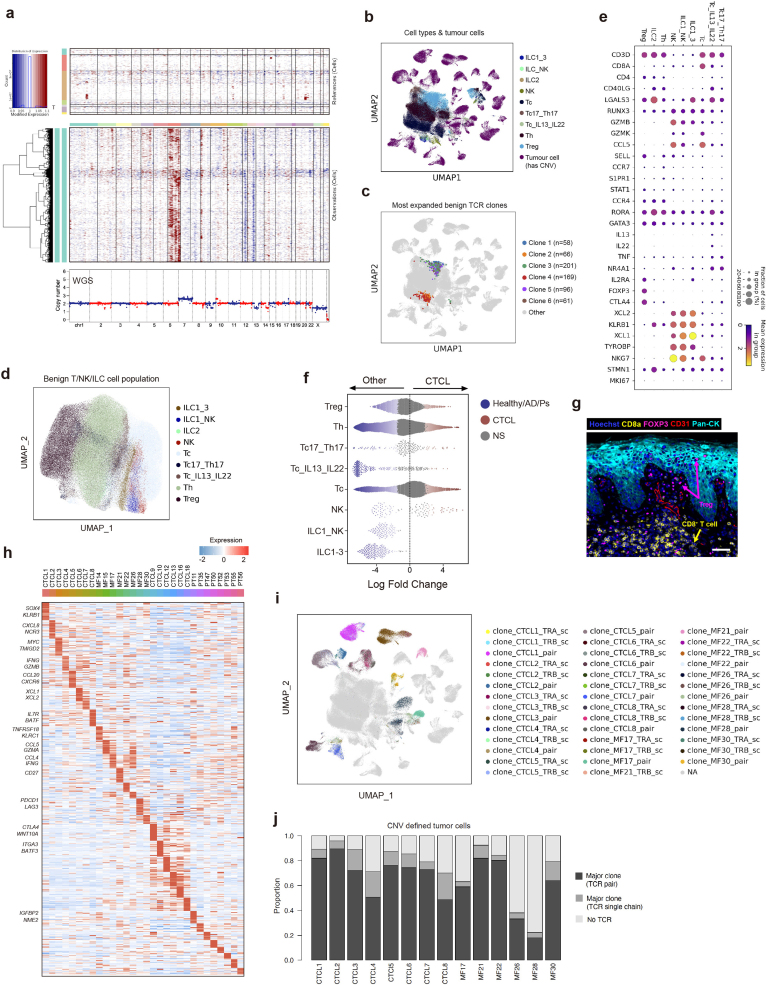
Malignant and benign T cells. **a**, Upper: Heat map showing CNVs inferred from scRNA-seq data in a representative sample (CTCL1). Top panel represents reference normal cells. Bottom heat map represents malignant T cells. Lower: CNVs inferred from whole genome sequencing (WGS) of the same sample. **b**, UMAP visualisation of the malignant T cell and benign T/NK/ILC cells in our CTCL dataset, coloured by cell types. **c**, UAMP of all T cells from CTCL skin showing the six most expanded benign T cell clones determined by TCR analysis in the dataset. The size of each expanded clonotype is in the parentheses. **d**, UMAP visualisation of benign T and NK/ILC populations in integrated CTCL and skin cell atlas data, coloured by cell types. **e**, Gene expression dot plot of marker genes for T cell and NK/ILC subsets. **f**, Beeswarm plot of the log-transformed fold changes in abundance of cells in CTCL versus those in healthy skin, AD and psoriasis from skin cell atlas. Differential abundance neighbourhoods at FDR 10% are coloured. NS, not significant. **g**, A multiplex protein immunofluorescence image (Rarecyte) in a representative tumour. Scale bar, 50 μm. Image representative of n = 8 patients (triplicate staining performed for each patient skin sample). **h**, Heat map showing inter-tumour DEGs across patients. Representative genes are labelled. **i**, UMAP of T, NK and ILC cells in our CTCL dataset coloured by the predominant TCR clonotype in each patient. ‘pair’ represents T cells that have both TCR alpha and beta chains. ‘TRA_sc’ represents cells that only have TCR alpha chain. ‘TRB_sc’ represents cells that only have TCR beta chain. **j**, Bar plot showing the proportion of CNV defined malignant T cells with the major expanded TCR clonotype alpha-beta pair, the major TCR clonotype single alpha or beta chain, and no TCR in each tumour.

To distinguish malignant/tumor cells from benign infiltrating T cells in CTCL skin, we inferred large-scale chromosomal copy number variations (CNVs) based on scRNA-seq data (Methods). As expected, malignant T cells exhibited extensive CNVs across their genomes consistent with those identified from whole-genome sequencing of the same tumor (Extended Data Fig. 2a). CNV inference could distinguish malignant clones from benign T cell clonal expansion in the TME as assessed by TCR clonality alone (Extended Data Fig. 2b,c).

**Fig. 3:**
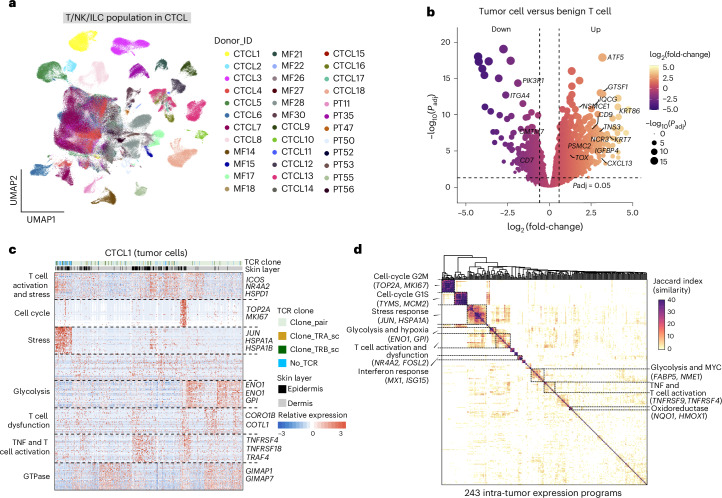
Characterization of malignant T cells in CTCL. **a**, UMAP visualization of T cells, NK cells and innate lymphoid cells (ILC) in our CTCL dataset. Dots are colored by donors. **b**, Volcano plot showing DEGs between malignant and benign T cells. Dot color indicates the log_2_(transformed fold-change). Dot size indicates the log_10_(transformed *P*_adj_ values). *P* values are calculated using a quasi-likelihood *F*-test and adjusted using the Benjamini–Hochberg procedure. **c**, Heat map showing gene expression programs and intratumor expression heterogeneity among malignant T cells in a representative patient. Program annotations and representative genes are labeled. The color key indicates scaled expression levels. **d**, Heat map depicting shared expression metaprograms across all patients. The Jaccard index is used to measure the similarity between any two intratumor expression programs.

Benign infiltrating T cells from different patients clustered together (Fig. 3a and Extended Data Fig. 2b). We identified nine T cell/natural killer (NK) cell/innate lymphoid cell subsets in CTCL, healthy skin, AD and psoriasis, showing different expression features (Extended Data Fig. 2d,e). Differential abundance testing revealed enrichment of regulatory T (T_reg_) cells, CD8^+^ T cells with a cytotoxic profile (T_c_ cells) and T_H_ cells in CTCL (Extended Data Fig. 2f,g). Overall, the composition of benign lymphocytes in CTCL resembles that found in the TME of cutaneous squamous and melanocytic cancers, with a high abundance of cytotoxic CD8^+^ T cells, potentially pro-tumorigenic CD4^+^ T_H_ cells and T_reg_ cells^30,31^.

In contrast to benign T cells, malignant T cell clones clustered by patient and displayed inter-patient gene expression heterogeneity (Fig. 3a and Extended Data Fig. 2b,h), for example, highly cytotoxic-like and upregulated *IFNG* and *GZMB* in patient CTCL4 (Extended Data Fig. 2h). Projection of dominant TCR clonotypes (from 14 patients) on to the uniform manifold approximation and projection (UMAP) revealed that malignant T cells from each patient almost exclusively harbored a single clonally expanded T cell receptor (Extended Data Fig. 2i), in keeping with published reports on CTCL tumor cells^9,16,21^. Notably, some malignant cells had lost either one or both TCR chains (Extended Data Fig. 2i,j), consistent with known loss of T cell phenotypic identity by histopathological staining and observed in scRNA-seq profiling^21^.

**Extended Data Fig. 3:**
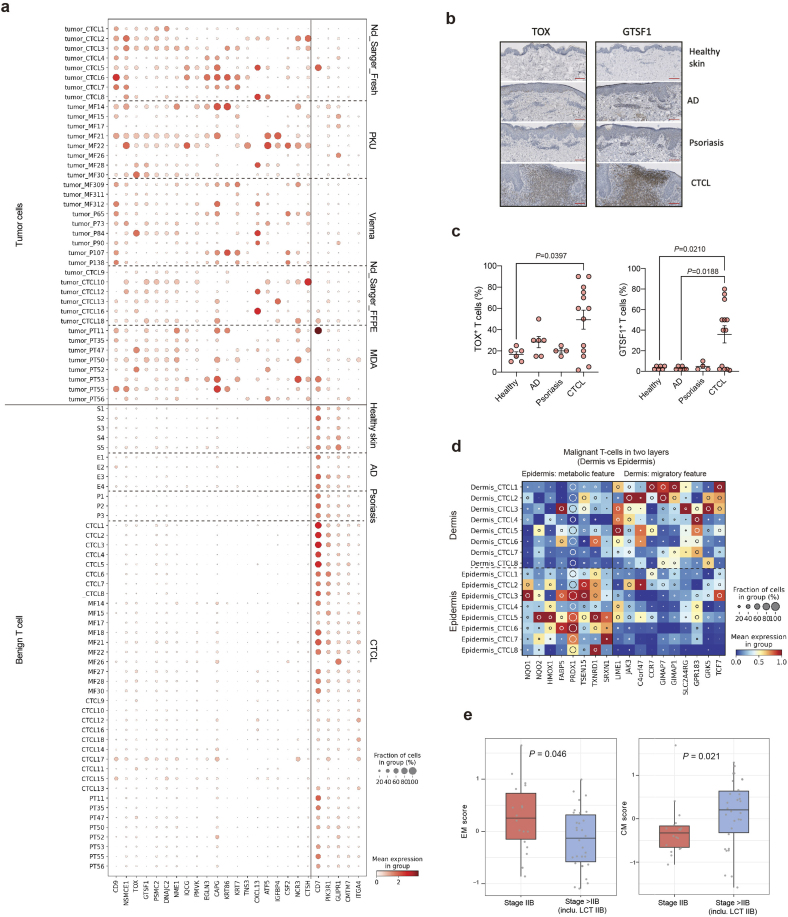
Characterisation of malignant T cells in CTCL. **a**, Gene expression dot plot of DEGs in malignant T cells across patients in the integrated CTCL datasets. **b**, IHC staining for TOX and GTSF1 in representative healthy skin, AD, psoriasis and CTCL samples. Scale bar, 100 µm. **c**, Dot plots comparing the IHC results in healthy skin (n = 6), AD (n = 6), psoriasis (n = 4) and CTCL samples (n = 13). *P* values are calculated using ordinary one-way ANOVA with Tukey correction for multiple testing. **d**, Heat map of DEGs between malignant T cells from dermis and epidermis. Colour represents expression level standardised between 0 and 1. The inset circle indicates the percentage of cells expressing a given gene. **e**, Box plots showing the expression of two gene scores in bulk RNA-seq data. Patients are divided into two groups: stage IIB (without large cell transformation: LCT; n = 18) and over stage IIB plus LCT (n = 30). EM, effector memory; CM, central memory. Gene lists for calculating scores are shown in Fig. 4a. *P* value is calculated using a two-sided Wilcoxon rank sum test. The lower edge, upper edge and centre of the box represent the 25th (Q1) percentile, 75th (Q3) percentile and the median, respectively. The interquartile range (IQR) is Q3 – Q1. Outliers are values beyond the whiskers (upper, Q3 + 1.5 × IQR; lower, Q1 − 1.5 × IQR).

To further distinguish the molecular properties of malignant T cells from benign infiltrating T cells in CTCL, we analyzed differentially expressed genes (DEGs) between malignant T cells and benign T cells from healthy skin, AD, psoriasis and CTCL. We identified previously reported features such as *TOX* upregulation and *CD7* downregulation^32,33^ (Fig. 3b, Extended Data Fig. 3a and Supplementary Table 2). Twenty-five genes provided good discriminatory power to distinguish malignant from benign T cells (Extended Data Fig. 3a). For instance, *CD9*, which encodes a cell-surface glycoprotein, was highly expressed in 27 out of 39 tumors (Extended Data Fig. 3a). It is interesting that we observed high expression of *CXCL13* in malignant T cells (Extended Data Fig. 3a), suggestive of a B cell homing and recruitment role^34^.

We validated protein expression of TOX and GTSF1, previously reported to distinguish malignant CTCL cells^35^, in lesional skin using IHC (*n* = 13) and observed increased, but highly variable, expression of these markers (Extended Data Fig. 3b,c). Although these upregulated genes in malignant T cells show potential as biomarkers, no marker alone could identify all CTCL tumors, demonstrating the heterogeneity of CTCL and the need to profile the presence of several genes or markers for diagnostic precision.

### Intratumor gene expression programs in malignant T cells

To dissect intratumoral malignant T cell heterogeneity in CTCL and identify features shared across all CTCL tumors, we analyzed intratumoral co-expressed gene modules (Fig. 3c). We identified 243 intratumor expression programs in total and classified nine metaprograms shared by subpopulations of malignant T cells in multiple tumors (Fig. 3d and Supplementary Table 3). We observed a stress-related metaprogram enriched for genes, such as *JUN* and *HSPA1A*, and two cell-cycle metaprograms (G1S and G2M), including *TOP2A* and *TYMS*, respectively. Two T cell activation-related metaprograms were characterized by the expression of genes such as *NR4R2, TNFRSF9* and *TNFRSF4*. A glycolysis- and hypoxia-related metaprogram was characterized by the expression of genes such as *ENO1* and *GPI* (Fig. 3d and Supplementary Table 3). This metabolic feature is shared by cancer cells in hypoxic environments and circulating memory T cells, but not tissue-resident T cells^36^, and could be explored in future studies on metabolic targets in CTCL.

Malignant T cells are known to exhibit epidermotropism in early disease but are more numerous in the dermis in advanced CTCL^37^. By sampling and profiling epidermal and dermal lesions separately in eight patients, we could compare gene expression between malignant T cells from both compartments to distinguish epidermotropic malignant T cells from their dermal counterparts. We found higher expression of cell migration-related genes including *CCR7* in dermal malignant T cells, in contrast to higher expression of metabolism-related genes like *NQO1/2*, *FABP5* and *PRDX1* in epidermal malignant T cells (Extended Data Fig. 3d and Supplementary Table 4).

### Malignant T cells in advanced-stage CTCL exhibit T_H_2 cell skewing

**Fig. 4:**
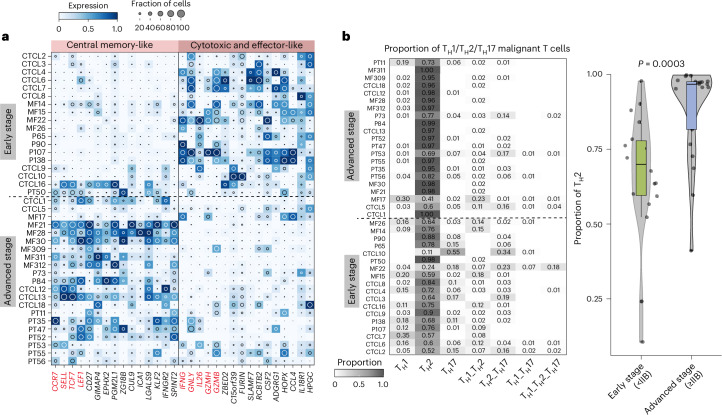
Comparison of malignant T cells from early stage and advanced-stage CTCL. **a**, Heat map of DEGs in early or advanced-stage CTCL samples. Color represents expression level categorized between 0 and 1. The inset circle indicates the percentage of cells expressing a given gene. **b**, Left, heat map showing the proportion of putative T_H_1, T_H_2 and T_H_17 cell-like malignant cells in each patient with CTCL. Patients are categorized into early or advanced stages. Right, violin plot comparing the proportion of T_H_2 cell-like malignant cells in early stage (*n* = 18) and advanced-stage (*n* = 21) CTCL samples. The *P* value was calculated using a two-sided Wilcoxon’s rank-sum test. The lower edge, upper edge and center of the box represent the 25th (Q1) percentile, 75th (Q3) percentile and median, respectively. The interquartile range (IQR) is Q3 − Q1. Outliers are values beyond the whiskers (upper, Q3 + 1.5 × IQR; lower, Q1 − 1.5 × IQR).

We next compared the gene expression profile between malignant T cells from early stage (<IIB stage) and advanced-stage disease (≥IIB stage). In early stage samples, malignant T cells were cytotoxic and tissue-resident effector like, as reflected by higher expression of *IFNG*, *GZMB* and *GNLY*, and lower expression of *CCR7*. By contrast, malignant T cells from advanced-stage disease expressed genes characteristic of central memory cells, including *SELL*, *CCR7*, *LEF1* and *TCF7* (Fig. 4a and Supplementary Table 5), suggesting increased capacity for malignant T cells to circulate in more advanced disease. This pattern was also observed when comparing early and advanced-stage CTCL bulk RNA-seq data (Extended Data Fig. 3e; both *P* < 0.05, two-sided Wilcoxon’s rank-sum test). Next, we determined the phenotype of malignant T cells based on the expression of the T cell lineage transcription factors *TBX21* (T_H_1 cells), *GATA3* (T_H_2 cells) and *RORC* (T_H_17 cells), and compared the proportion of T helper cell types across disease severity. Compared with early stage disease, a significantly higher proportion of T_H_2 cell-like malignant T cells was observed in advanced-stage disease (Fig. 4b; *P* = 0.0003, two-sided Wilcoxon’s rank-sum test), suggesting either T_H_2 cell skewing of T_H_1 cell-/T_H_17 cell-like malignant cells or preferential survival of T_H_2 cell-like malignant cells with disease progression.

### MHC-II^+^ fibroblasts probably support malignant cells in CTCL

**Fig. 5:**
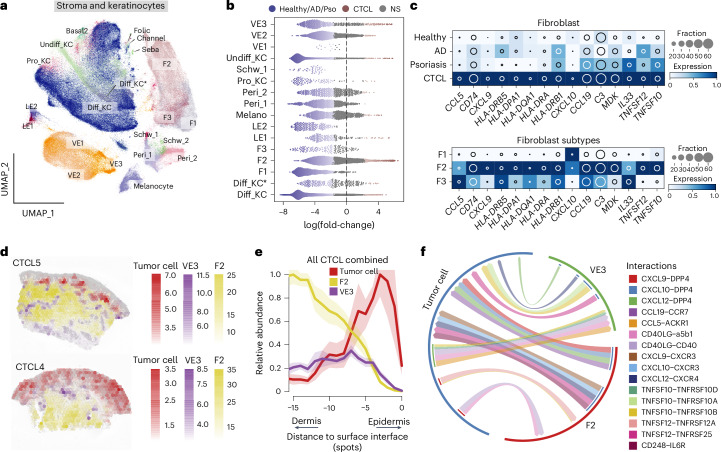
Characterization of the stromal and keratinocyte compartments in CTCL. **a**, UMAP visualization of the stromal and keratinocyte (KC) population in the current dataset integrated with skin cell atlas, colored by cell types. **b**, Beeswarm plot of the log-transformed fold-changes in abundance of stromal cell subsets in CTCL versus those in healthy skin, AD and psoriasis. Differential abundance neighborhoods at FDR of 10% are colored. **c**, Heat map showing gene expression in CTCL-enriched fibroblasts. The color represents the expression level standardized between 0 and 1. The inset circle indicates the percentage of cells expressing a given gene. **d**, Spatial mapping of F2, VE3 and tumor cells in two representative samples. The estimated abundance (color intensity) is overlaid on histology images. **e**, Curve plot showing the mean (across all samples) per-spot normalized abundance of F2, VE3 and tumor cells along the axis to skin surface. Shaded regions represent the 95% 2 s.d. confidence intervals (CIs). **f**, Circos plot showing putative ligand–receptor interactions between F2 or VE3 and malignant T cells. Representative interactions are colored. Diff, Differentiated; F, fibroblasts; Melano, melanocytes; Schw, Schwann.

**Extended Data Fig. 4:**
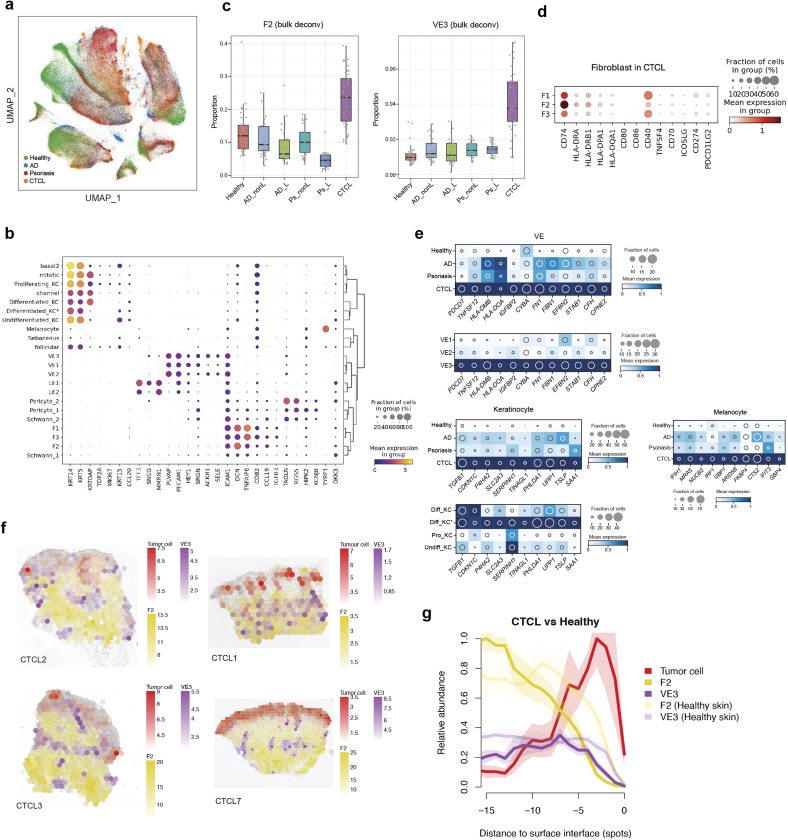
Characterisation of stromal population in CTCL and comparing with healthy skin, AD and psoriasis. **a**, UMAP visualisation of the stromal population in the current dataset integrated with skin cell atlas, coloured by skin conditions. **b**, Gene expression dot plot of marker genes for stromal cell subsets. Dot colour indicates log-transformed and normalised expression value. Dot size indicates the percentage of cells in each cell type expressing a given gene. **c**, Box plot of deconvolution of F2 and VE3 in bulk RNA-seq datasets of healthy skin (n = 38), AD (nonlesion: n = 27; lesion: n = 27), psoriasis (nonlesion: n = 27; lesion: n = 28) and CTCL (n = 49). The lower edge, upper edge and centre of the box represent the 25th (Q1) percentile, 75th (Q3) percentile and the median, respectively. The interquartile range (IQR) is Q3 – Q1. Outliers are values beyond the whiskers (upper, Q3 + 1.5 × IQR; lower, Q1 − 1.5 × IQR). **d**, Dot plot showing MHC-II and co-stimulatory molecules expression in fibroblasts in CTCL. **e**, Heat maps of DEGs in CTCL enriched keratinocyte, vascular endothelial cell and melanocyte. Colour represents expression level standardised between 0 and 1. The inset circle indicates the percentage of cells expressing a given gene. **f**, Spatial mapping of F2, VE3 and tumour cells in four representative CTCL samples. Estimated abundance (colour intensity) is overlaid on histology images. **g**, Curve plot showing the mean (across all samples) per-spot normalised abundance of F2, VE3 (in healthy and CTCL skin) and tumour cells along the axis to skin surface. Shaded regions represent the 95% 2 SD confidence intervals.

Using our integrated scRNA-seq dataset from CTCL, healthy, AD and psoriasis skin^22^, we compared the stromal cell and keratinocyte compartments across these conditions. Subclustering of these cells identified 22 cell subtypes in CTCL, healthy, AD and psoriasis skin (Fig. 5a and Extended Data Fig. 4a,b). Differential cellular abundance analysis revealed the greatest enrichment in fibroblast subtype F2 and vascular endothelial subtype VE3 in CTCL (Fig. 5b and Extended Data Fig. 4a). We performed deconvolution analysis of bulk RNA-seq datasets^23,24^ using BayesPrism^38^, which validated the enrichment of F2 and VE3 (Extended Data Fig. 4c; all *P* < 10^−4^, two-sided Wilcoxon’s rank-sum test).

To understand the function of CTCL-enriched fibroblasts and vascular endothelial cells, we compared DEGs between CTCL and the other three conditions (Methods and Supplementary Table 6). It is interesting that we found that antigen presentation MHC-II genes (for example, *CD74*, *HLA-DRB5* and *HLA-DPA1*) were upregulated in CTCL-enriched fibroblasts but in the absence of co-stimulatory gene (*CD86* and *CD80*) expression, in line with observations in pancreatic cancer^39^ (Fig. 5c and Extended Data Fig. 4d). Upregulation of MHC-II genes was predominant in CTCL-enriched F2 cells that also expressed chemokine genes *CCL5*, *CXCL9* and *CXCL10* and genes of T_H_2 cell-promoting cytokines such as *IL33* (Fig. 5c). The F2 CTCL skin fibroblasts resemble previously reported MHC-II^+^ antigen-presenting fibroblasts in several cancer types including pancreatic adenocarcinoma and breast cancer^39,40^, MHC-II^+^ lymph node T cell-zone reticular cells (TRCs) expressing CXCL9^41^ and TLS fibroblasts^42^. Furthermore, F2 fibroblasts transcriptionally resemble fetal skin fibroblasts^22^, lending support to previous reports on co-optation of developmental cell states in inflammatory disease^22^ and cancer^43^. MHC-II genes were also upregulated in CTCL-enriched vascular endothelial cells (Extended Data Fig. 4e).

Spatial transcriptomics-based mapping between F2, VE3 and malignant T cells were in keeping with the expected location of F2 in the dermis and malignant T cells in the epidermis in early stage CTCL (Fig. 5d,e and Extended Data Fig. 4f). The spatial distribution of F2 and VE3 in healthy and CTCL skin is similar (Extended Data Fig. 4g). As a result of the transcriptional resemblance between CTCL MHC-II^+^ fibroblasts and lymphoid organ TRCs and their spatial overlap with malignant T cells in the dermis (Extended Data Fig. 1h), we hypothesized that MHC-II^+^ fibroblasts may be interacting with and promoting malignant T cell growth in CTCL, analogous to naive T cells receiving MHC-II^+^ antigen-presenting cell (APC) survival support in the lymph node^44,45^. We therefore inferred intercellular communications between the F2, VE3 and malignant T cells in CTCL based on putative ligand–receptor interactions. Our analysis predicted putative cell–cell interactions between F2, VE3 and malignant T cells via ligand receptors such as TNFSF12–TNFRSF25, CXCL9–DPP4 and CD40–CD40LG (Fig. 5f). These observations suggest that MHC-II^+^ fibroblasts probably support CTCL tumors in lesional skin. Furthermore, we also observed an enrichment of keratinocytes expressing *TSLP*, well recognized to promote T_H_2 cell microenvironment in AD^46^, in CTCL skin (Extended Data Fig. 4e).

### APCs in CTCL TME promote T cell activation and T_H_2 cell skewing

**Fig. 6:**
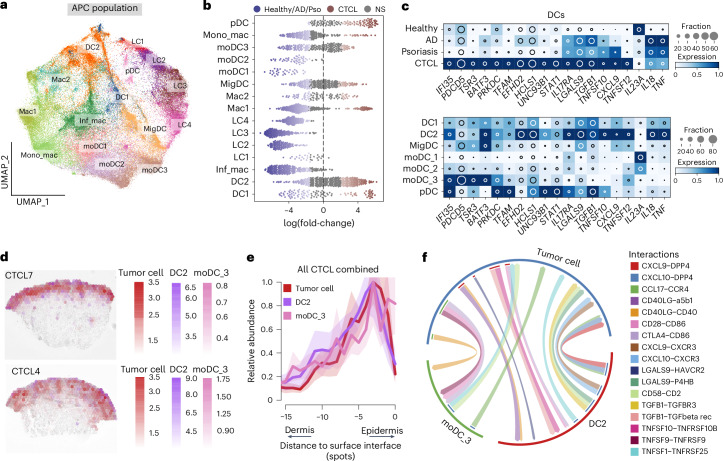
Characterization of the APC population in CTCL. **a**, UMAP visualization of the APC population in the current dataset integrated with skin cell atlas, colored by cell types. **b**, Beeswarm plot of the log-transformed fold-changes in abundance of stromal cell subsets in CTCL versus those in healthy skin, AD and psoriasis. Differential abundance neighborhoods at FDR of 10% are colored. **c**, Heat map showing gene expression in CTCL-enriched DCs. The color represents expression level standardized between 0 and 1. The inset circle indicates the percentage of cells expressing a given gene. **d**, Spatial mapping of DC2, moDC_3 and tumor cells in two representative samples. Estimated abundance (color intensity) is overlaid on histology images. **e**, Curve plot showing the mean (across all samples) per-spot normalized abundance of DC2, moDC_3 and tumor cells along the axis to the skin surface. Shaded regions represent the 95% 2 s.d. CIs. **f**, Circos plot showing putative ligand–receptor interactions. Representative interactions are colored.

**Extended Data Fig. 5:**
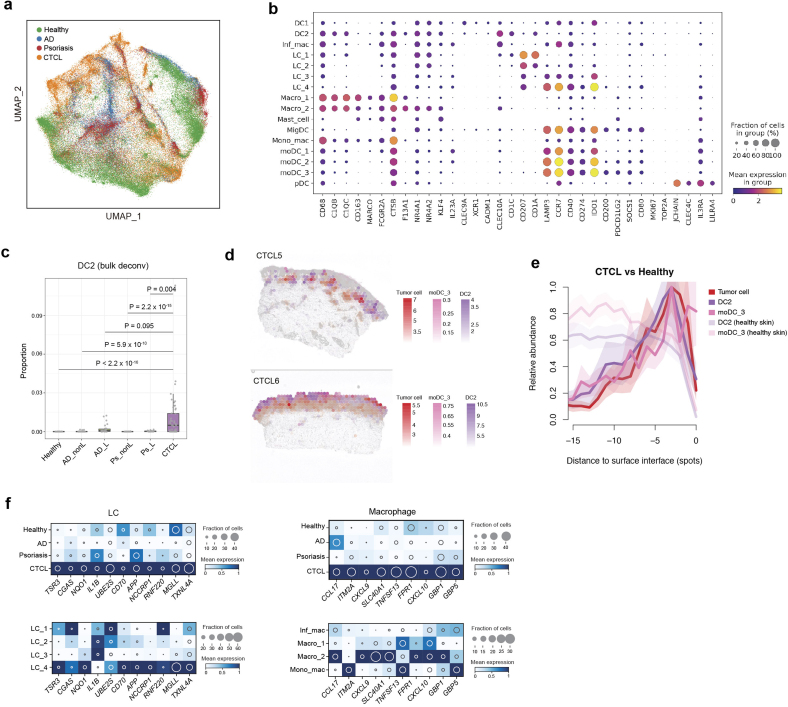
Characterisation of APC population in CTCL and comparing with healthy skin, AD and psoriasis. **a**, UMAP visualisation of the APC population in the current dataset integrated with skin cell atlas, coloured by skin conditions. **b**, Gene expression dot plot of marker genes for APC cell subsets. Dot colour indicates log-transformed and normalised expression value. Dot size indicates the percentage of cells in each cell type expressing a given gene. **c**, Box plot of deconvolution of DC2 in bulk RNA-seq datasets of healthy skin (n = 38), AD (nonlesion: n = 27; lesion: n = 27), psoriasis (nonlesion: n = 27; lesion: n = 28) and CTCL (n = 49). Two-sided Wilcoxon rank-sum. The lower edge, upper edge and centre of the box represent the 25th (Q1) percentile, 75th (Q3) percentile and the median, respectively. The interquartile range (IQR) is Q3 – Q1. Outliers are values beyond the whiskers (upper, Q3 + 1.5 × IQR; lower, Q1 − 1.5 × IQR). **d**, Spatial mapping of moDC_3, DC2 and tumour cells in two representative samples. Estimated abundance (colour intensity) is overlaid on histology images. **e**, Curve plot showing the mean (across all samples) per-spot normalised abundance of DC2, moDC_3 (in healthy and CTCL skin) and tumour cells along the axis to skin surface. Shaded regions represent the 95% 2 SD confidence intervals. **f**, Heat maps of DEGs in CTCL enriched Langerhans cell (LC) and macrophage. Colour represents expression level standardised between 0 and 1. The inset circle indicates the percentage of cells expressing a given gene.

To investigate whether CTCL skin APCs support malignant T cells, we performed subclustering of the APC population and annotated different subsets based on the skin cell atlas data^22^ (Fig. 6a and Extended Data Fig. 5a,b). Using differential abundance testing, we found several DC subsets including moDC_3 and DC2 to be substantially enriched in CTCL compared with healthy skin, AD and psoriasis (Fig. 6b). The enrichment of DC2 can also be found in bulk RNA-seq data (Extended Data Fig. 5c). It is interesting that CTCL-enriched DCs showed higher expression of Galectin *LGALS9* and the gene encoding the tumor necrosis factor (TNF) ligand *TNFSF12*, molecules known to negatively regulate T_H_1 cell immunity^47^ and mediate tumor immune responses^48^ (Fig. 6c). We observed spatial proximity between DC2, moDC_3 and epidermotropic malignant T cells in CTCL skin (Fig. 6d,e). Notably, DC2 and moDC_3 in CTCL were enriched in the epidermis in contrast to their locations in the dermis in healthy skin (Fig. 6d,e and Extended Data Fig. 5d,e). We further predicted cellular interactions between DC2, moDC_3 and malignant T cells via the ligand–receptor pairs CD58–CD2, which have previously been reported to mediate cancer immune evasion^49^, and putative interactions via LGALS9–HAVCR2 (also known as TIM3), LGALS9–P4HB and TNFSF12–TNFRSF25 (Fig. 6f).

We compared other APC subsets in CTCL with their counterparts in healthy skin, AD and psoriasis (Extended Data Fig. 5f). CTCL-derived Langerhans cells showed higher expression of the co-stimulatory molecule *CD70* (Extended Data Fig. 5f). Notably, genes related to T_H_1 and T_H_17 cell skewing (for example, *IL23A* and *IL18*) were downregulated in DCs in CTCL skin (Fig. 6c), further supporting a T_H_2 cell-permissive, malignant T cell microenvironment.

### CTCL lesional B cells interact with malignant T cells

**Fig. 7:**
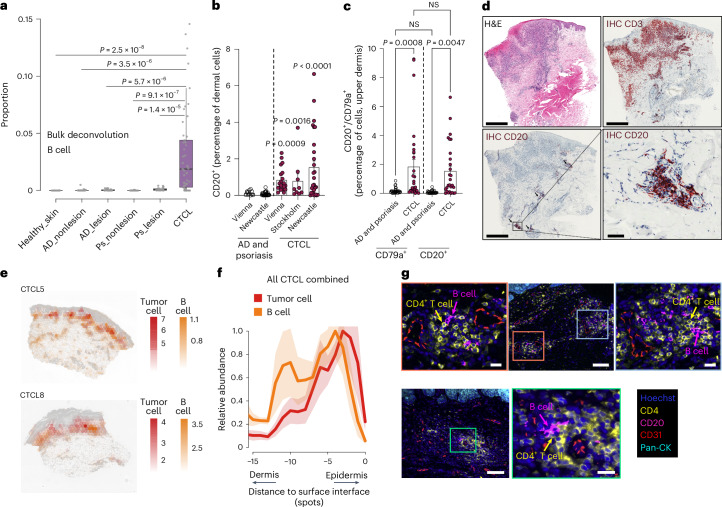
B cell enrichment in CTCL. **a**, Box plot showing deconvolution of B cells in bulk RNA-seq datasets of healthy skin (*n* = 38), AD (nonlesion: *n* = 27; lesion: *n* = 27), psoriasis (Ps; nonlesion: *n* = 27; lesion: *n* = 28) and CTCL (*n* = 49). The numbers of samples in the categories are labeled. The *P* value is calculated using a two-sided Wilcoxon’s rank-sum test. The lower edge, upper edge and center of the box represent the 25th (Q1) percentile, 75th (Q3) percentile and median, respectively. The IQR is Q3 − Q1. Outliers are values beyond the whiskers (upper, Q3 + 1.5 × IQR; lower, Q1 − 1.5 × IQR). **b**, Bar plot showing IHC staining of CD20 in AD, psoriasis and CTCL skin samples in three independent cohorts (AD or psoriasis cohorts, *n* = 27, *n* = 15, respectively; CTCL cohorts, *n* = 27, 9, 20). The *P* values were calculated using ordinary one-way analysis of variance (ANOVA) with Tukey’s correction for multiple testing. The error bars show the s.e.m. **c**, Bar plot showing CD79a^+^ and CD20^+^ cells in CTCL (*n* = 27) and AD or psoriasis (*n* = 30). The *P* values were calculated using ordinary one-way ANOVA with Tukey’s correction for multiple testing. Data are shown as individual values and the mean percentage of IHC-positive cells among all cells of the dermis; error bars indicate the s.e.m. **d**, H&E image and IHC staining for CD3 and CD20 in a representative sample. The zoomed-in box and arrows highlight B cells. Scale bars, 1 mm and 200 μm (zoomed-in). **e**, Spatial mapping of B cells and tumor cells in two representative samples. The estimated abundance (color intensity) is overlaid on the histology images. **f**, Curve plot showing the mean (across all samples) per-spot normalized abundance of B cells and tumor cells along the axis to the skin surface. Shaded region represents the 95% 2 s.d. CIs. **g**, Multiplex protein immunofluorescence images (RareCyte) in two representative tumors. Representative views of B cell and CD4^+^ T cell interaction are zoomed in. Scale bars, 100 μm and 20 μm (zoomed-in). Images from two patients, representing *n* = 8 patients (triplicate staining performed for each patient skin sample).

**Extended Data Fig. 6:**
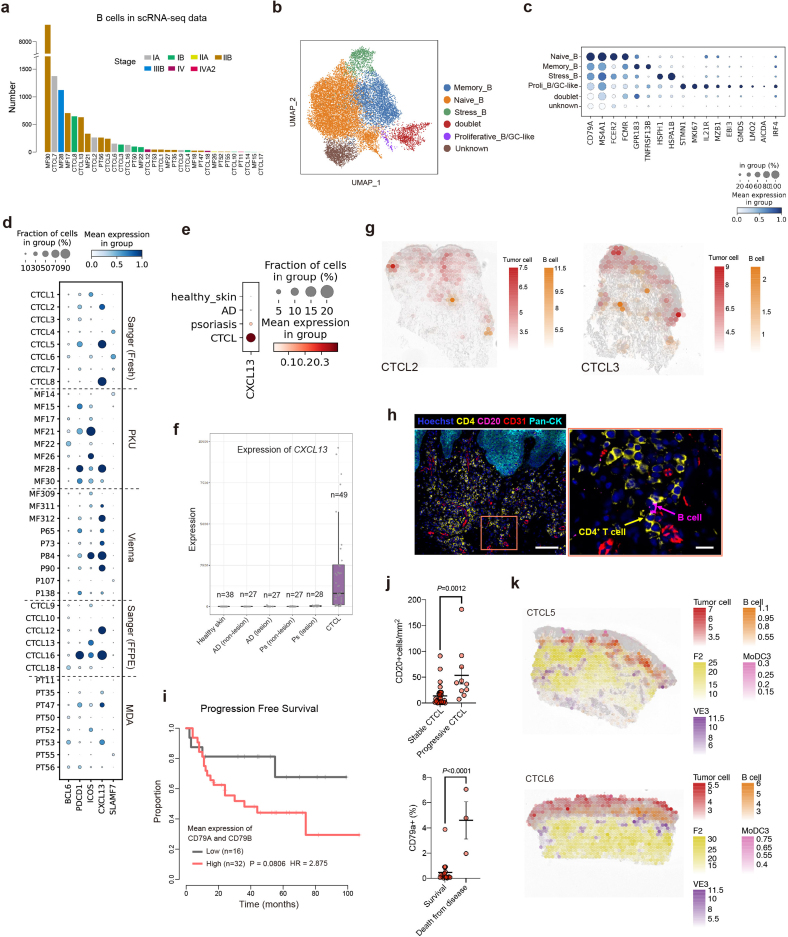
B cell enrichment in CTCL. **a**, Bar plot showing number of B cells from patients captured by scRNA-seq. Colours indicate different disease stages. **b**, UMAPs of B cell population in our dataset coloured by cell subtypes. **c**, Gene expression dot plot of marker genes for B cell subsets. Dot colour indicates log-transformed and normalised expression value. Dot size indicates the percentage of cells in each cell type expressing a given gene. **d**, Dot plot showing the expression of follicular T cell marker genes in malignant T cells in three scRNA-seq datasets. **e**, Dot plot showing the expression of CXCL13 among the T cell population in healthy skin, AD, psoriasis and CTCL. **f**, Box plot showing the expression of *CXCL13* in bulk RNA-seq data from healthy skin (n = 38), AD (nonlesion: n = 27; lesion: n = 27), psoriasis (nonlesion: n = 27; lesion: n = 28) and CTCL samples (n = 49). The lower edge, upper edge and centre of the box represent the 25th (Q1) percentile, 75th (Q3) percentile and the median, respectively. The interquartile range (IQR) is Q3 – Q1. Outliers are values beyond the whiskers (upper, Q3 + 1.5 × IQR; lower, Q1 − 1.5 × IQR). **g**, Spatial mapping of B cell and tumour cells in two representative samples. Estimated abundance (colour intensity) is overlaid on histology images. **h**, A multiplex protein immunofluorescence image (Rarecyte) in a representative tumour. A representative view showing B cell and CD4 + T cell interaction is zoomed in. Scale bars, 100 μm and 20 μm. Image representative of n = 8 patients (triplicate staining performed for each patient skin sample). **i**, Progression free survival probability of CTCL patients according to stratification of B cell abundance estimated by mean expression of CD79A and CD79B. HR, hazard ratio. *P* value is calculated by multivariate Cox regression. **j**, IHC staining of CD20 and CD79a in stable and progressive CTCL skin samples, and survival and death cases. Data shown as individual values of CD20+ cells per mm2 +/− SEM and mean percentages of CD79a+ cells among all cells +/− SEM, n = 27 (Vienna cohort). A two-sided unpaired t-test is used. **k**, Spatial mapping of tumour cells, F2, VE3, moDC_3, DC2 and B cell in Visium data for two representative tumours. Estimated abundance for cell types (colour intensity) across locations (dots) is overlaid on histology images.

B cells, sometimes organized in lymphoid structures, have been reported in several cancer TMEs where they can prime and stimulate anti-tumor T cells and produce tumor-directed antibodies^50^. We therefore sought to confirm our earlier observation of increased B cell abundance in CTCL skin in a larger cohort and investigate whether B cells were present as aggregates within lymphoid structures in CTCL lesional skin. First, we confirmed that B cells were present in most patients with CTCL in our scRNA-seq data, regardless of disease stage (Extended Data Fig. 6a). Then, we assessed whether the B cell enrichment is evident in a larger CTCL patient cohort. Using bulk deconvolution analysis (*n* = 196), we confirmed significantly greater proportions of B cells present in CTCL skin samples compared with healthy skin, lesional and nonlesional AD and psoriatic skin (Fig. 7a; all *P* < 10^−4^, two-sided Wilcoxon’s rank-sum test). We further validated the increased presence of B cells in CTCL skin biopsies using IHC by staining for CD20 and CD79a in three independent CTCL cohorts (*n* = 56) (Fig. 7b,c).

Notably, we found that B cells formed aggregates in 55% (31 out of 56) of CTCL IHC samples (Fig. 7d). The aggregates detected in our IHC samples were reminiscent of early TLSs, which usually contain germinal center (GC) B cells. We therefore subclustered the B cell population in our CTCL dataset and identified several subsets including naive and memory B cells (Extended Data Fig. 6b,c). It is interesting that we identified GC-like B cells expressing *EBI3*, *GMDS* and *LMO2* (Extended Data Fig. 6c). In addition, we detected the expression of genes associated with follicular helper T (T_FH_) cells and B cell recruitment (for example, *BCL6*, *PDCD1* and *CXCL13*) in malignant T cells (Extended Data Fig. 6d). *CXCL13* was highly and almost specifically expressed in CTCL compared with healthy skin, AD and psoriasis (Extended Data Fig. 6e,f), probably explaining the enrichment of B cells in CTCL. CTCL fibroblasts also share expression features with TRCs in healthy lymph nodes^41^, which may indicate a role in TLS formation.

**Fig. 8:**
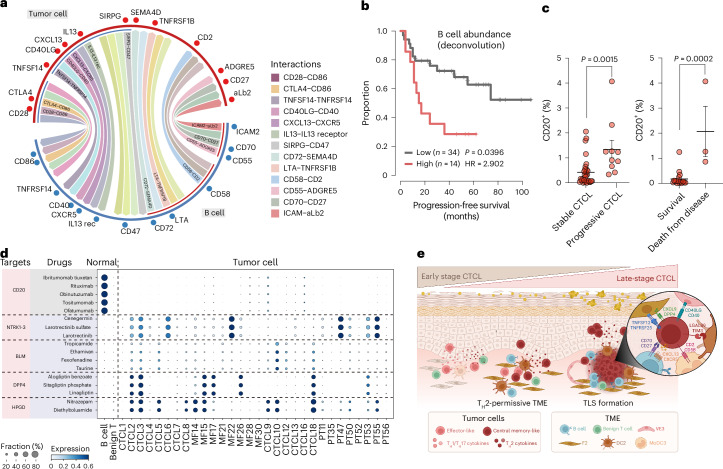
B cells interact with malignant T cells and associate with prognosis. **a**, Circos plot showing putative ligand–receptor interactions between B cells and malignant T cells. Representative interactions are colored. **b**, Progression-free survival probability of patients with CTCL according to stratification of B cell abundance estimated by bulk deconvolution. The *P* value was calculated by multivariate Cox’s regression. **c**, IHC staining of CD20 in stable and progressive CTCL skin samples and survival and death cases. Data shown as mean percentages of CD20^+^ cells per mm^2^ ± s.e.m. (*n* = 27 (Vienna cohort)). A two-sided, unpaired Student’s *t*-test was used. **d**, Dot plot showing the expression of drug targets predicted by drug2cell^51^. **e**, Schematic of the features depicting the TME of CTCL. Panel **e** created in BioRender. Strobl, J. (2024) BioRender.com/q48l570.

Spatial transcriptomics and multiplex protein immunofluorescence imaging showed proximity and direct cell–cell contact between CD20^+^ B cells and CD4^+^ tumor cells in CTCL TME (Fig. 7e–g and Extended Data Fig. 6g,h). We next performed cell–cell interaction analysis and identified putative ligand–receptor interactions between B cells and tumor cells, which included genes for co-stimulatory interaction partners such as CD70–CD27, CD40LG–CD40, CD58–CD2 and CD28–CD86, which are known to promote T cell activation and B cell recruitment interaction, CXCL13–CXCR5 supporting lymphoid structure formation (Fig. 8a).

Collectively, these observations suggest a role for B cells in promoting tumor growth and we hypothesized that B cell abundance in CTCL skin would correlate with disease severity and clinical outcome. The abundance of B cells, inferred from bulk RNA-seq data, was indeed associated with poor disease prognosis (Fig. 8b and Extended Data Fig. 6i). In accordance with this, we detected increased percentages of B cells in progressive CTCL skin lesions using IHC (Fig. 8c and Extended Data Fig. 6j). It is of interest that three patients from the Vienna cohort, who died from CTCL, displayed increased B cell presence in tumor samples (IIB stage at biopsy) taken 3–7 years before death (Fig. 8c and Extended Data Fig. 6j). These findings support the utility of B cell immunostaining as a potential diagnostic and prognostic aid for CTCL, particularly in early stage disease.

We next performed drug2cell^51^ analysis to predict potential therapeutic targets and identified several known CD20-directed antibodies (rituximab and obinutuzumab; Fig. 8d and Supplementary Table 7). Our data provide evidence for B cells as a therapeutic target in CTCL, in alignment with isolated reports of patients with CTCL responding to incidental or intentional treatment with the B cell-depleting agent rituximab^52,53^. In addition, drug2cell analysis also identified 15-hydroxyprostaglandin dehydrogenase as a potential selective drug target against malignant T cells, while sparing benign T cells.

Together, our data revealed a trajectory of malignant T cells to co-opt T_H_2 cell-like gene programs that appear to be supported by a T_H_2 cell-permissive, pro-tumorigenic TME in CTCL (Fig. 8e and Extended Data Fig. 6k). The T_H_2 cell CTCL TME was associated with B cells forming aggregates and TLSs that correlated with disease progression and poor clinical outcome (Figs. 7d and 8b and Extended Data Fig. 6i).

## Discussion

CTCL exhibits a wide spectrum of genetic and clinical alterations with limited specific histological features in the early stage, impeding diagnosis. Our findings revealed clonal T_H_2 cell-like malignant cells in a T_H_2 cell-permissive, tumor-promoting microenvironment mainly contributed by MHC-II^+^ fibroblasts and DCs. T_H_2 cell-like malignant T cells are also associated with B cell infiltration and aggregate formation, which could be used to aid CTCL diagnosis and potentially treatment. Other cell types in the TME of CTCL and their potential functions warrant further investigation in future studies.

Our in-depth characterization of malignant T cell clones revealed extensive CNVs across their genomes. Despite high inter-patient heterogeneity, which had also been reported in previous CTCL transcriptome studies^9,16,21^, we found gene expression metaprograms that were shared by different tumors. In addition to these metaprograms, we identified features that were not ubiquitous among all tumors, but could be collectively adopted for diagnostic use. This included the DNA-binding protein TOX, which has been proposed as a potential but not exclusive marker for CTCL^54^, and GTSF1, a protein with a function in T cells that is unknown and previously identified in some CTCL cancer cell lines^55^, and the presence of B cells in CTCL skin. Importantly, our analyses revealed that B cell abundance is associated with progressive disease and poor prognosis. We noted the need for future functional studies to confirm the pathogenic role of B cells in driving CTCL TLS formation and disease progression. Nevertheless, our observations of potential TLS formation in CTCL are supported by previous dermatopathological studies that identified the expression of T_FH_ cell markers in CTCL^56^.

Importantly, benign B cell infiltration in CTCL needs to be distinguished from rare cases of mycosis fungoides-type CTCL showing aberrant expression of CD20 in malignant T cells^57^. The presence of B cell aggregates in the CTCL TME could become a useful diagnostic aid to distinguish CTCL from nonmalignant skin diseases and as a potential therapeutic target. There have been reports of patients with CTCL who responded after treatment with rituximab for another clinical reason^52^ or as an off-label indication^58^. Collectively, our data suggest that B cell-depleting therapies may effectively target the CTCL TME, providing evidence for larger clinical trials to assess B cell depletion as a therapeutic avenue for CTCL. In addition, the interaction that we identified between malignant T cells and moDC_3 or DC2 via the CCL17–CCR4 axis may also explain the therapeutic efficacy of mogamulizumab in CTCL^17^.

A T_H_2 cell TME has been found to foster tumor growth in nonhematopoietic solid tumors including breast and pancreatic cancer^59,60^. In the present study, we showed that malignant T cells in CTCL co-opt a T_H_2 cell-immune program to promote recruitment of B cells and TLSs in the skin, a nonlymphoid tissue. The T_H_2 microenvironment may, in turn, promote the survival of malignant T cells. Whether the T_H_2 cell-immune program deployment is aided by an antigen-specific (including response to a skin microbe) or antigen-nonspecific process remains to be determined. It is interesting that the blocking antibody to the IL-4 receptor, dupilumab, has been shown to unmask (CTCL misdiagnosed as AD) or worsen CTCL symptoms^61^, possibly by increasing free IL-4 and IL-13 to bind to the IL-13α2 receptor^62,63^.

In summary, our findings provide a new understanding of CTCL malignant cells within their TME, including the co-optation of a T_H_2 cell-immune program resulting in B cell aggregates and TLS formation in advanced-stage disease. These findings provide evidence to support the deployment of B cell-directed (combination) therapies to treat patients with CTCL.

## Methods

### Patient recruitment and sample acquisition

CTCL skin samples generated for the present study were donated with written consent and approval from the Newcastle and North Tyneside NHS Health Authority Joint Ethics Committee (protocol 08/H0906/95+5). Each patient donated two skin-punch biopsies, from a representative plaque or tumor. One biopsy was used for scRNA-seq and the other for bulk sequencing and IHC. All patients had mycosis fungoides, diagnosed based on correlation of clinical and histopathological features. The stage of CTCL at the time of the biopsy was based on clinical assessment performed by dermatology specialists at the Department of Dermatology and National Institute for Health and Care Research (NIHR) Newcastle Biomedical Research Centre. For fixed sample acquisition for both IHC and single-cell flex, FFPE blocks were accessed through the Newcastle CEPA Biobank (17/NE/0070). For additional IHC validation cohorts, samples were donated with consent from the local ethics committee at the Medical University of Vienna (ECS 1360/2018) and the Swedish Ethical Review Authority (2019-03467). CTCL diagnosis and staging as well as monitoring for disease progression were performed by specialists in dermatology and dermatohistopathology at the Department of Dermatology, Medical University of Vienna and the Department of Dermatology, Karolinska University Hospital, Stockholm. Detailed patient information including sex and age is provided in Supplementary Table 1.

### Fresh skin sample processing

Skin biopsies in eight patients were immediately processed by removing the lower dermis and subcutis and separating the epidermis and dermis after dispase II digestion at a concentration of 2 U ml^−1^ for 2–3 h at 37 °C. The epidermis and dermis were processed separately in type IV collagenase at a concentration of 1.6 mg ml^−1^ overnight (37 °C, 5% CO_2_). Subsequently, single-cell suspensions were formed by vigorous pipetting and filtering (100-μm filter), counted and further processed via FACS.

### FACS sorting and 10x Genomics Chromium loading

Cells from both the epidermis and the dermis were stained with an antibody panel containing CD45 (BUV395, clone HI30, BD Biosciences, 563792), CD8a (Alexa Fluor-700, clone HIT8a, BioLegend, 300920) and sorted using FACS into the following fractions: CD45^−^, CD45^+^CD8a^+^ and CD45^+^CD8a^−^. FlowJo was used to analyze FACS data. A target of 10,000 cells was used to calculate the loading volume for the 10x Chromium, taken from the manufacturer’s protocol (10x Genomics User Guide, CG000207). Each fraction sorted from the epidermis and dermis was loaded on to one channel of the 10x Chromium chip before running on the Chromium Controller using the Chromium Next GEM Single Cell 5′ v.1.1 kit.

### Library preparation and sequencing

Gene expression libraries were generated from the resulting complementary DNA after clean-up following the 10x Genomics protocols. Enriched TCR cDNA was also generated from each CD45^+^ fraction (10x Genomics Chromium Single Cell V(D)J Enrichment Kit, Human T Cell) and subsequent libraries were made. All libraries were sequenced using an Illumina NovaSeq with the gene expression libraries sequenced to achieve a minimum of 50,000 reads per cell and the T libraries sequenced to achieve a minimum of 5,000 reads per cell.

### Single-cell fixed RNA profiling

CTCL FFPE sample blocks were utilized for single-cell fixed RNA profiling using the single-cell gene expression flex kit from 10x Genomics. Sixteen samples from fourteen patients were sectioned using a microtome (Leica, RM2235) and, for each sample, four sections were cut at 25-μm thickness. Sample sections were deparaffinized and pestle dissociated according to the manufacturer’s guidelines (10x Genomics User Guide, CG000632) and stored short term at 4 °C. Four sample pools, each containing four samples, were generated by multiplexing and probe hybridized for 18 h, followed by gel beads-in-emulsion generation, barcoding and library preparation according to the 10x Genomics user guide (CG000527) and sequenced down one lane of a Novaseq 6000 S4 flow cell.

### Whole-genome sequencing

DNA was extracted from frozen skin samples in RNA Later (Invitrogen) using the AllPrep Micro kit (Qiagen, 80284) following the manufacturer’s protocol. The DNA was quantified using a Qubit with the High Sensitivity DNA kit (Invitrogen, Q32851). Library preparation was carried out using NEBNext Ultra II DNA Library Prep Kit from Illumina. Libraries were uniquely dual indexed to mitigate tag hopping, quantified, equimolar pooled and sequenced on an Illumina NovaSeq 6000 platform (150-bp paired-end reads).

### FFPE Visium CytAssist spatial transcriptomics

RNA quality and tissue morphology of CTCL FFPE sample blocks were assessed before FFPE Visium processing. Each sample block was sectioned using a microtome (Leica, RM2235) at 5-μm thickness on to a SuperFrost Plus microscope slide (VWR, 6310108), incubated for 3 h at 42 °C, dried overnight in a desiccator at room temperature and processed for FFPE Visium within 2 weeks of sectioning. Deparaffinization, hematoxylin and eosin (H&E) staining and decrosslinking steps were performed as per the manufacturer’s recommendations (10x Genomics Demonstrated Protocol, CG000520) and sections were imaged on a Hamamatsu Nanozoomer. Sections were then further processed with FFPE Visium CytAssist v.2 chemistry (6.5-mm) kit and dual-indexed libraries were prepared as per the 10x Genomics User Guide (CG000495). Four libraries were pooled at a time and sequenced down one lane of an Illumina Novaseq SP flow cell with the following run parameters: read 1: 28 cycles; i7 index: 10 cycles; i5 index: 10 cycles; read 2S: 50 cycles.

### Fresh frozen Visium spatial transcriptomics

Healthy skin samples were embedded fresh into Optimal Cutting Temperature embedding medium and frozen using an isopentane-dry ice slurry. All tissues were sectioned at a thickness of 15 µm and a tissue optimization experiment using Visium Spatial Gene Expression Reagent Kits—Tissue Optimization (10x Genomics) was carried out to determine the optimum permeabilization time (14 min) using the manufacturer’s protocol. Spatial gene expression libraries were then generated using the Visium Spatial Gene Expression Reagent Kits (10x Genomics) and the manufacturer’s protocol and sequenced using an Illumina NovaSeq 6000 to achieve a minimum number of 50,000 read pairs per tissue-covered spot. H&E images needed for analysis were taken using a Zeiss AxioImager with an apotome microscope (Carl Zeiss Microscopy) and Brightfield imaging (Zeiss Axiocam 105 48-color camera module) at ×20 magnification. The ZEN blue edition v.3.1 (Carl Zeiss Microscopy) software was then used to acquire the images and adjust the *z*-plane and light balance, as well as stitching the image tiles to retrieve the overall H&E image file.

### RareCyte 16-plex immunofluorescence staining

FFPE sample blocks were sectioned using a microtome (Leica RM2235) at 5-µm thickness. Slides were dried at 60 °C for 60 min to ensure adherence. After deparaffinization, sections were subjected to antigen retrieval for 5 min at 95 °C followed by 5 min at 107 °C using EZ-AR 2 Elegance AR buffer (pH 8.5; BioGenex, HK547-XAK). To remove autofluorescence, slides were bleached with AF Quench Buffer which consists of 4.5% H_2_O_2_/24 mM NaOH in phosphate-buffered saline (PBS) and quenched for 60 min with a strong white light exposure (using the HIGH setting) followed by further quenching for 30 min using an ultraviolet transilluminator (365-nm HIGH setting). Slides were rinsed with 1× PBS and incubated in 300 µl of Image-iT FX Signal Enhancer (Thermo Fisher Scientific, I36933) for 15 min. After rinsing with surfactant wash buffer (0.025% Triton X-100 in 1× PBS), the slides were stained with 300 µl of labeled primary antibodies in mouse/rabbit diluent (PBS antibody stabilizer (CANDOR Bioscience, 131500) containing 5% mouse serum and 5% rabbit serum) for 120 min. All antibodies (Supplementary Table 8) were pre-diluted according to the manufacturer’s recommendations. Slides were washed with a surfactant wash buffer and incubated with 300 µl of Hoechst 33342 (Thermo Fisher Scientific, H3570) in goat diluent (10% goat serum in PBS antibody stabilizer) for 30 min, after washing in 1× PBS. Finally, slides were coverslipped using ArgoFluor mounting medium (RareCyte, 241301000) and imaged on the following day using a RareCyte Orion microscope with a ×20 objective. Scans were performed using Imager and relevant acquisition settings were applied using the software Artemis.

### scRNA-seq data processing

We processed newly generated and published raw scRNA-seq data using a consistent pipeline. In brief, raw sequencing data for published datasets (National Center for Biotechnology Information (NCBI) accession HRA000166 and Sequence Read Archive (SRA) accession PRJNA754592) were downloaded from the National Genomics Data Center (NGDC) and the SRA, respectively. For HRA000166, we downloaded raw data for both gene expression and V(D)J because they are publicly available. For accession PRJNA754592, only gene expression raw data were available, because the authors of the original study submitted only bam files from which V(D)J fastqs cannot be restored. As a result, V(D)J data from accession PRJNA754592 were not included. Gene expression data from droplet-based sequencing were processed using STARsolo^64^ (v.2.7.10a_alpha_220818) and the 2020-A reference genome. STARsolo outputs were then processed with cellbender^65^ (v.0.2.1) to remove ambient RNA contamination. For the flex samples, CellRanger (v.8.0.0) was used for cell calling with Chromium_Human_Transcriptome_Probe_Set_v1.0.1_GRCh38-2020-A as a reference. We initially had 16 samples from 14 patients. Samples with <400 cells were removed, so there were finally 11 flex samples from 10 patients. V(D)J data were processed using the 10x software package CellRanger vdj (v.7.2.0) and the ‘refdata-cellranger-vdj-GRCh38-alts-ensembl-7.1.0′ reference genome. Gene expression outputs from STARsolo were read using the read_10x_mtx function in Scanpy^66^ (v.1.8.1 and v.1.9.3). Data objects from different 10× lanes were then concatenated using the concatenate function in anndata (v.0.7.6). To detect and remove doublets, we applied Scrublet^67^ (v.0.2.3) to the data from each 10× lane to obtain per-cell scrublet scores and used a doublet exclusion threshold of median plus four median absolute deviations of the doublet score, as previously described^22^. Cells with >20% mitochondrial gene expression or expression of <400 detected genes, or total gene counts <1,000, were excluded from downstream analysis. Genes that were expressed in fewer than three cells were also removed.

### Data clustering and integration

After preprocessing and quality control of the data, we created two major data objects: (1) CTCL plus skin cell atlas object, and (2) CTCL-only object through data integration. To create the first data object, we integrated the newly generated CTCL scRNA-seq data (from both fresh and FFPE samples) with CTCL data from accessions HRA000166 and PRJNA754592 and healthy skin, AD and psoriasis data from an existing skin cell atlas^22^. The integration was carried out using the scVI module in scvi-tools^68^ (v.0.20.3) as the following steps. The highly variable genes were selected by sc.pp.highly_variable_genes (n_top_genes=4000, batch_key=‘donor’, span=1). The model was built with (n_hidden=100, n_layers=1, n_latent=6, gene_likelihood=‘zinb’), using batch_key=’donor’ and categorical_covariate_keys= [‘technology’] (10x versus Flex). The integrated object was clustered by Leiden clustering at a resolution of 1. To broadly annotate cell types, gene expression counts were normalized and log(transformed). The expression of main cell markers was visualized on the main object and clusters were annotated accordingly. For the granular annotation, we used CellTypist to train a classifier model based on annotations from the skin cell atlas^22^ and then used the model to annotate the integrated object. However, as the model does not include B cells because they are not in the skin cell atlas data, the B cell cluster was manually annotated based on marker gene expression. To make the second data object, we integrated the newly generated CTCL scRNA-seq data (from both fresh and FFPE samples) with CTCL data from accessions HRA000166 and PRJNA754592. The top 4,000 highly variable genes among donors were selected using sc.pp.highly_variable_genes function in scanpy. Then scVI model was set up with donor as batch key and study and technology as technical covariates. The following parameters were used for the model: n_hidden=100, n_layers=1, n_latent=5, gene_likelihood=‘zinb’. As all CTCL samples are already part of the first object (with skin atlas samples), annotations were transferred from that object to the CTCL object.

### Differential abundance analysis using Milo

To reveal potential differences in cellular abundance in CTCL, we performed differential abundance analysis comparing CTCL with healthy skin, AD and psoriasis using milopy, a python implementation of Milo^27^. For the overall integrated object, we first performed a random subsampling using the subsample function in Scanpy, which subsampled the overall object to 0.2 of the total number of cells. Then the standard milopy pipeline was run for the data object with the number of neighbors (n_neighbors) being set to 100 and the proportion of graph vertices to randomly sample (prop in the milo.make_nhoods function) being set to 0.05. For the APCs, stromal and benign cell populations, we did not subsample the objects and set n_neighbors and prop to 100 and 0.05, respectively. Beeswarm plots were made using the plotDAbeeswarm function to show the log(transformed fold-changes) in abundance of cells in CTCL versus those in healthy skin, AD and psoriasis for each data object.

### Inferring CNVs based on scRNA-seq data

To effectively distinguish malignant T cells and nonmalignant cells, we inferred large-scale chromosomal CNVs of single cells based on scRNA-seq data using the tool InferCNV (https://github.com/broadinstitute/inferCNV) with default parameters. In brief, InferCNV first orders genes according to their genomic positions (first from chromosome 1 to chromosome X and then by gene start position) and then uses a previously described sliding-average strategy to normalize gene expression levels in genomic windows with a fixed length. Multiple putative nonmalignant cells are chosen as the reference to further denoise the CNV result. From the CNV inference, we did not detect malignant T cells in six patients, which is consistent with the report from a study that we integrated^21^.

### Intratumor expression programs and metaprograms

To explore intratumor expression programs, we applied non-negative factorization (implemented in the R NMF package) to the tumor cells from each patient. In brief, for each tumor, we first normalized the expression counts using the NormalizeData function in Seurat with default parameter settings. Highly variable genes (HVGs) were then selected using the FindVariableFeatures function in Seurat^69^. Next, we performed center scale for HVGs and regressed out the percentage of mitochondrial genes using the ScaleData function. For NMF analysis, all negative values in the expression matrix were replaced by 0. The top ten ranked NMF gene modules in each tumor sample were extracted using the nmf function in the NMF package. For each gene module, we extracted the top 30 genes with the highest weight, which were used to define a specific intratumor expression program. Finally, we included only intratumor expression programs that had s.d. values >0.1 among tumor cells. To investigate whether some intratumor expression programs were actually shared by multiple tumors, we applied a clustering analysis to all programs based on the pair-wised Jaccard index calculated as follows, where A and B represent two intratumor programs:

$${\rm{Jaccard\; index}}={\rm{A}}\bigcap {\rm{B}}/{\rm{A}}\bigcup {\rm{B}}.$$

We defined those intratumor programs shared by multiple tumors as metaprograms. We interpreted metaprograms by checking functions of individual genes and based on the 3CA database^70^ (https://www.weizmann.ac.il/sites/3CA).

### Subclustering and annotation of different cell compartments

We performed subclustering and annotation of different cell compartments based on the object integrated with the skin cell atlas data. Cells were subsetted into stromal and keratinocyte, APC and benign T cell, NK cell and innate lymphoid cell populations. To improve separation of subclusters for the stromal and keratinocyte and APC populations, we reintegrated the cells to new embeddings. The top 2,000 and 1,000 HVGs were selected, respectively. To create new embeddings, an scVI model was set up with the donor as batch key, technology as technical covariate and the default scVI model parameters. We used the labels transferred from the skin cell atlas using CellTypist to annotate cells. These objects were then used to analyze differential abundance of granular cell types between CTCL and other conditions.

For the B cell compartment, as most B cells are from CTCL samples, we subsetted them from the CTCL object. HVGs were detected using the highly_variable_genes function in scanpy with minimum cut-off values of 0.0125 and 0.5 for expression and dispersion. We excluded immunoglobulin genes from HVGs when running principal component analysis. To correct the batch effect, we used the harmonypy^71^ package and set the donor as the batch key with the θ value being set to 3.

### Visium processing and spatial mapping with cell2location

Sequencing reads from 10x Genomics Visium FFPE libraries were aligned to the human transcriptome reference GRCh38-2020-A using 10x Genomics SpaceRanger (v.2.1.0) and exonic reads were used to produce messenger RNA count matrices for each sample. The 10x Genomics SpaceRanger was also used to align paired histology images with mRNA capture spot positions in the Visium slide. To spatially map the cell types annotated in scRNA-seq data to their spatial locations in tissues, we applied cell2location to integrate scRNA-seq CTCL data and Visium FFPE mRNA count matrices as described previously^28^. Briefly, the cell2location model estimates the abundance of each cell type in each location by decomposing mRNA counts in Visium FFPE data using the transcriptional signatures of reference cell types derived from scRNA-seq data. Two major steps were in the analysis using cell2location: (1) we applied a negative binomial regression model implemented in cell2location and estimated the reference signature of fine-grained annotated cell types in integrated scRNA-seq data from CTCL and healthy samples. In this step, we used an unnormalized mRNA count matrix as input and filtered it to 12,711 genes and 626,529 cells. Donor IDs were regarded as the batch category, donor, study and 10x version as covariates, and the following parameters were used to train the model: ‘max_epochs’ = 250, ‘batch_size’ = 2,500, ‘train_ size’ = 1 and ‘Ir’ = 0.002. (2) The reference signature model was further used by cell2location to estimate spatial abundance of cell types. We kept genes that were shared with scRNA-seq and estimated the abundance of cell types in the Visium data from 8 CTCL samples (from 8 patients) and 15 healthy skin samples (from 7 donors). In this step, cell2location was used with the following parameter settings: training iterations = 50,000, number of cells per location *N* = 30, ‘detection_alpha’ = 20. We further performed NMF of the cell-type abundance estimates from cell2location to identify the spatial co-occurrence of cell types with ‘n_fact’ being set to 5–30. For downstream analysis, considering that our Visium samples consisted of a single piece of tissue, we removed all spots that correspond to tissue debris (which are all spot groups except the largest one). We considered outermost epidermis spots as skin surface and used them to calculate Euclidean distances from each spot to the closest surface spot and expressed this distance in interspot distances (100 μm). To estimate cell-type abundance dependent on distance to surface, we first normalized cell-type abundance by dividing it by per-spot totals. Then, we grouped spots by rounded distance to surface and calculated the mean and s.d. for each cell type and each distance.

### DEG analysis using a pseudo-bulk strategy

We applied a pseudo-bulk strategy to the analysis of DEGs across (1) malignant and benign T cells, (2) malignant T cells from epidermis and dermis, (3) malignant T cells from early stage and advanced-stage samples, and (4) microenvironmental cells from CTCL and the other three conditions. In brief, we aggregated raw counts of each gene by donor and used donors rather than cells as biological replicates. DEG analyses were carried out using R package edgeR^72^. For the analysis of (1), we excluded nonlesion cells from AD and psoriasis and regarded healthy skin, AD and psoriasis as one comparator (other). We filtered genes by expression levels using the filterByExpr function in edgeR with ‘min.count’ and ‘min.total.count’ being set to default. We designed the model matrix using the model.matrix function and included only one variable, namely groups (malignant T cell and benign T cell). For the analysis of (2), we first divided malignant T cells from the eight patients with separate sampling of epidermis and dermis into those from epidermis and dermis, and conducted pseudo-bulk analysis with both tissue layer and derived patient being considered (for example, CTCL1_dermis and CTCL1_epidermis were aggregated separately). We filtered genes by expression levels using the filterByExpr function in edgeR with ‘min.count’ and ‘min.total.count’ being set to 2 and 10, respectively. In the model matrix design, we fit the model on paired samples considering both tissue (dermis and epidermis) and patient (CTCL1–CTCL8). For the analysis of (3), we included both dataset (Peking University (PKU), MD Anderson Cancer Center (MDA), Vienna, Ncl_Sanger_Fresh and Ncl_Sanger_FFPE) and group (early stage and late stage) as variables to consider variation across studies. For the analysis of (4), we included 10× CTCL data from fresh samples (both newly generated and published) because the probe-based 10x Flex for FFPE samples does not capture MHC-II genes. We excluded nonlesion cells from AD and psoriasis and regarded healthy skin, AD and psoriasis as one comparator (other). We excluded samples with <20 cells of a specific cell type. We filtered genes by expression levels using the filterByExpr function in edgeR, with ‘min.count’ and ‘min.total.count’ being set to default. We designed the model matrix using the model.matrix function and included only one variable, namely groups CTCL and other (healthy skin, AD and psoriasis). For all the analysis, we fit genewise, negative binomial, generalized linear models with quasi-likelihood tests using the glmQLFit and glmQLFTest functions in edgeR.

### Bulk deconvolution of cell types

For bulk deconvolution analysis, we first downloaded published bulk RNA-seq datasets of healthy skin, AD, psoriasis, and CTCL from the Gene Expression Omnibus (GEO) database with accessions GSE121212 and GSE168508. A single-cell reference for deconvolution analysis was then prepared by excluding tumor cells and randomly downsampling the integrated object (healthy skin, AD, psoriasis and CTCL) to 8% of total cells. BayesPrism^38^ was used for deconvolution analysis with raw counts for both single-cell and bulk RNA-seq data as inputs. Both the ‘cell-type labels’ and the ‘cell-state labels’ were set to fine-grained annotations. Ribosomal protein genes and mitochondrial genes were removed from single-cell data because they are not informative in distinguishing cell types and can be a source of large spurious variance. We also excluded genes from sex chromosomes and lowly transcribed them as recommended by the BayesPrism tutorial. For further analysis, we applied a pairwise Student’s *t*-test to select DEGs with ‘pval.max’ being set to 0.01 and ‘lfc.min’ to 0.1. Finally, a prism object containing all data required for running BayesPrism was created using the new.prism() function, and the deconvolution was performed using the run.prism() function. Two-sided Wilcoxon’s rank-sum test was performed to examine any statistically significant enrichment.

For the survival analysis, the CTCL bulk RNA-seq cohort was grouped into high and low abundance of B cells (both estimated by bulk deconvolution and mean expression of *CD79A* and *CD79B*) by the optimal cut point determined using the cutp() function in the survMisc R package. We performed multivariate analyses using Cox’s proportional hazards model (coxph() function in the survival R package) to correct clinical covariates including age, gender and tumor stage for the survival analysis. Kaplan–Meier survival curves were plotted to show differences in survival time using the ggsurvplot() function in the survminer R package.

### Inference of cell–cell interactions

We inferred potential cell–cell interactions using CellPhoneDB^73^ (v.4). In brief, we randomly downsampled the CTCL object to 100 cells per fine-grained cell type per donor. The generated object was then used to run CellPhoneDB analysis with default parameters and thresholds. For the downstream visualization, we used the plot_cpdb3 function in the R package ktplots (https://github.com/zktuong/ktplots).

### Prediction of druggable targets using drug2cell

To predict potential druggable targets on B cells and malignant T cells, we ran drug2cell^51^ on these two cell types together with benign T cells as a comparator. Drug2cell is druggable target prediction tool that integrates drug–target interactions from the ChEMBL database (https://www.ebi.ac.uk/chembl) with single-cell data to comprehensively evaluate drug target expression in single cells. We first calculated per-cell scores of ChEMBL drug targets using the d2c.score() function. Then, we performed differentially expressed analysis on ChEMBL drugs by comparing B cells, benign T cells and malignant T cells using the scanpy tl.rank_genes_groups() function. When visualizing the result, we separated malignant T cells by patients to show drugs that potentially function in multiple patients, given the strong inter-patient heterogeneity of CTCL tumors.

### IHC of FFPE samples for TOX and GTSF1

IHC staining for TOX and GTSF1 was performed on skin samples from healthy skin, AD, psoriasis and CTCL. In addition to the skin samples collected for scRNA-seq, a further cohort of patients with CTCL gave informed written consent for previous clinical samples to be used. Automated IHC staining was performed by NovoPath on FFPE slides using the Ventana Discovery Ultra autostainer (Roche) and the DISCOVERY ChromoMap DAB Kit. Antibodies used for staining were anti-GTSF1 (polyclonal, Atlas Antibodies, HPA038877) and anti-TOX (polyclonal, Atlas Antibodies, HPA018322). Scoring for TOX and GTSF1 was performed manually by a hematopathologist and dermatologist, reviewing the slides and deciding on an agreed approximation of positive staining. Identification of neoplastic T cells was based on their location, size and immunophenotype.

### IHC of FFPE samples for B cells

IHC staining was performed on FFPE skin samples of CTCL tumors. Skin biopsies were fixed in 10% neutral buffered formalin (Newcastle and Stockholm cohorts) or 4% formalin (Vienna cohort), then moved to 70% ethanol, dehydrated and embedded in paraffin. For tissues from Vienna, FFPE samples were cut into 4-μm sections, deparaffinized using a Neoclear (Sigma-Aldrich) and ethanol series and autoclaved in citrate buffer at pH 6.1 (Dako) to achieve antigen retrieval. Blocking with hydrogen peroxide was performed. Subsequently, slide tissue sections were subjected to automated IHC staining (Autostainer, Dako Agilent) using anti-CD20 antibody (clone L26, Dako, M0755) or anti-CD79a antibody (clone JCB117, Dako, M7050) followed by visualization (EnVision FLEX, Dako Omnis, Agilent). For samples from Newcastle, 4-µm FFPE sections were stained for CD20 or CD79a on the Ventana Benchmark Ultra automated staining platform with CC1 antigen retrieval and Ultraview-DAB detection. For analyses in the Stockholm cohort, FFPE samples were cut into 3.5-µm sections, deparaffinized and subjected to citrate buffer at pH 9 (Dako) to achieve antigen retrieval. Staining was manually performed using anti-CD20 antibody (clone EP459Y, Abcam, ab78237) and secondary goat anti-rabbit immunoglobulin G H&L antibody (Abcam, ab214880).

Stained sections were imaged and digitized with NanoZoomer S360 (Hamamatsu), Scanscope CD2 (Aperio Technologies), Zeiss AxioScan.Z1 Slide Scanner and TissueFAXS scanning system (TissueGnostics). Image-based automated cell detection for all samples was performed with HistoQUEST software (TissueGnostics).

### Statistics and reproducibility

For representative images from IHC and RareCyte, each experiment was repeated two to three times independently with similar results. Information on statistical tests is included in the figure legends.

### Reporting summary

Further information on research design is available in the Nature Portfolio Reporting Summary linked to this article.

## Online content

Any methods, additional references, Nature Portfolio reporting summaries, source data, extended data, supplementary information, acknowledgements, peer review information; details of author contributions and competing interests; and statements of data and code availability are available at 10.1038/s41590-024-02018-1.

## Supplementary information

Reporting Summary

Supplementary Tables Supplementary Tables 1–8.

## Data Availability

There are no restrictions on data availability for newly generated data presented in the present study. FASTQ files of all raw sequencing data from the present study have been deposited at EMBL-EBI ArrayExpress and are made publicly available at accessions E-MTAB-14559, E-MTAB-12303 and E-MTAB-13614. Previously published scRNA-seq datasets are available in the Genome Sequence Archive for humans (accession HRA000166), the NCBI BioProject database (accession HRA000166), ArrayExpress (accession E-MTAB-8142) and the GEO database (accession GSE173205). Previously published bulk RNA-seq datasets are available in the GEO database under accessions GSE168508 and GSE121212. Scanpy h5ad objects for CTCL, CCL plus skin cell atlas and Visium data are available for download and can be explored on an online web portal: https://collections.cellatlas.io/ctcl.

## Code availability

The code generated during the present study is available at GitHub: https://github.com/ruoyan-li/Cutaneous-T-cell-lymphoma-study.
