# Author Correction: Cutaneous T cell lymphoma atlas reveals malignant T_H_2 cells supported by a B cell-rich tumor microenvironment

**DOI:** 10.1038/s41590-024-02046-x

**Published:** 2024-12-02

**Authors:** Ruoyan Li, Johanna Strobl, Elizabeth F. M. Poyner, Aya Balbaa, Fereshteh Torabi, Pavel V. Mazin, Nana-Jane Chipampe, Emily Stephenson, Ciro Ramírez-Suástegi, Vijaya Baskar Mahalingam Shanmugiah, Louis Gardner, Bayanne Olabi, Rowen Coulthard, Rachel A. Botting, Nina Zila, Elena Prigmore, Nusayhah H. Gopee, Marta A. Chroscik, Efpraxia Kritikaki, Justin Engelbert, Issac Goh, Hon Man Chan, Harriet F. Johnson, Jasmine Ellis, Victoria Rowe, Win Tun, Gary Reynolds, Dexin Yang, April Rose Foster, Laure Gambardella, Elena Winheim, Chloe Admane, Benjamin Rumney, Lloyd Steele, Laura Jardine, Julia Nenonen, Keir Pickard, Jennifer Lumley, Philip Hampton, Simeng Hu, Fengjie Liu, Xiangjun Liu, David Horsfall, Daniela Basurto-Lozada, Louise Grimble, Chris M. Bacon, Sophie C. Weatherhead, Hanna Brauner, Yang Wang, Fan Bai, Nick J. Reynolds, Judith E. Allen, Constanze Jonak, Patrick M. Brunner, Sarah A. Teichmann, Muzlifah Haniffa

**Affiliations:** 1 Wellcome Sanger Institute, Wellcome Genome Campus, Hinxton, UK; 2 Department of Systems Biology, The University of Texas MD Anderson Cancer Center, Houston, TX USA; 3 MD Anderson UTHealth Graduate School of Biomedical Sciences, Houston, TX USA; 4 Department of Dermatology, Medical University of Vienna, Vienna, Austria; 5 Biosciences Institute, Newcastle University, Newcastle, UK; 6 Department of Dermatology and NIHR Newcastle Biomedical Research Centre, Newcastle, Hospitals NHS Foundation Trust, Newcastle upon Tyne, UK; 7 NovoPath, Department of Cellular Pathology, Newcastle Hospitals NHS Foundation Trust, Newcastle upon Tyne, UK; 8 Section Biomedical Science, University of Applied Sciences FH Campus Wien, Vienna, Austria; 9 Center for Immunology and Inflammatory Diseases, Massachusetts General Hospital, Boston, MA USA; 10 Division of Dermatology, Department of Medicine, Solna and Center for Molecular Medicine, Karolinska Institutet, Stockholm, Sweden; 11 Translational and Clinical Research Institute, Newcastle University, Newcastle upon Tyne, UK; 12 Biomedical Pioneering Innovation Center and School of Life Sciences, Peking University, Beijing, China; 13 Department of Dermatology and Venerology, Peking University First Hospital, Beijing, China; 14 Wolfson Childhood Cancer Research Centre, Translational and Clinical Research Institute, Newcastle University, Newcastle upon Tyne, UK; 15 Department of Cellular Pathology, Newcastle upon Tyne Hospitals NHS Foundation Trust, Newcastle upon Tyne, UK; 16 Department of Dermatology, Karolinska University Hospital, Stockholm, Sweden; 17 Lydia Becker Institute of Immunology and Inflammation, School of Biological Sciences, Faculty of Biology, Medicine and Health, Manchester Academic Health Science Centre, University of Manchester, Manchester, UK; 18 Department of Dermatology, Icahn School of Medicine at Mount Sinai, New York, NY USA; 19 Cambridge Stem Cell Institute, Jeffrey Cheah Biomedical Centre, Cambridge Biomedical Campus, University of Cambridge, Cambridge, UK; 20 Department of Medicine, University of Cambridge, Cambridge, UK

**Keywords:** Tumour immunology, Tumour immunology, Sequencing

Correction to: *Nature Immunology* 10.1038/s41590-024-02018-1, published online 18 November 2024.

In the version of the article initially published, the department was incorrect in affiliation 10 which has now been amended to the Division of Dermatology, Department of Medicine, Solna and Center for Molecular Medicine, Karolinska Institutet, Stockholm, Sweden in the HTML and PDF versions of the article.

