# Supplementary material for: Cutaneous T cell lymphoma atlas reveals malignant TH2 cells supported by a B cell-rich tumor microenvironment
Source: Nat Immunol. 2024 Nov 18;25(12):2320–30. doi: 10.1038/s41590-024-02018-1 (PMC11588665; doi:10.1038/s41590-024-02018-1)
Supplement: Supplementary file 1 — Reporting Summary [file 41590_2024_2018_MOESM1_ESM.pdf]

nature portfolio

Corresponding author(s): Muzlifah Haniffa
Sarah Teichmann
Ruoyan Li

Last updated by author(s): Oct 8, 2024

# Reporting Summary

Nature Portfolio wishes to improve the reproducibility of the work that we publish. This form provides structure for consistency and transparency in reporting. For further information on Nature Portfolio policies, see our Editorial Policies and the Editorial Policy Checklist.

## Statistics

For all statistical analyses, confirm that the following items are present in the figure legend, table legend, main text, or Methods section.

| n/a | Confirmed | |
|---|---|---|
| ☐ | ☒ | The exact sample size (*n*) for each experimental group/condition, given as a discrete number and unit of measurement |
| ☐ | ☒ | A statement on whether measurements were taken from distinct samples or whether the same sample was measured repeatedly |
| ☐ | ☒ | The statistical test(s) used AND whether they are one- or two-sided<br>*Only common tests should be described solely by name; describe more complex techniques in the Methods section.* |
| ☐ | ☒ | A description of all covariates tested |
| ☐ | ☒ | A description of any assumptions or corrections, such as tests of normality and adjustment for multiple comparisons |
| ☐ | ☒ | A full description of the statistical parameters including central tendency (e.g. means) or other basic estimates (e.g. regression coefficient) AND variation (e.g. standard deviation) or associated estimates of uncertainty (e.g. confidence intervals) |
| ☐ | ☒ | For null hypothesis testing, the test statistic (e.g. *F*, *t*, *r*) with confidence intervals, effect sizes, degrees of freedom and *P* value noted<br>*Give P values as exact values whenever suitable.* |
| ☒ | ☐ | For Bayesian analysis, information on the choice of priors and Markov chain Monte Carlo settings |
| ☒ | ☐ | For hierarchical and complex designs, identification of the appropriate level for tests and full reporting of outcomes |
| ☒ | ☐ | Estimates of effect sizes (e.g. Cohen's *d*, Pearson's *r*), indicating how they were calculated |

*Our web collection on statistics for biologists contains articles on many of the points above.*

## Software and code

Policy information about availability of computer code

| | |
|---|---|
| Data collection | No software was used for data collection. Software used for data alignment, quantification, quality control and downsteam analysis of single cell datasets in this study are described in detail in manuscript 'Methods'. |

| | |
|---|---|
| Data analysis | Single-cell sequencing data were processed and analysed using publicly available software packages. Further information on analytical approaches used in the study are further detailed in manuscript 'Methods' section.<br><br>Software and packages versions used for computational analysis are as follows:<br>STARsolo (version v2.7.10a_alpha_220818); CellRanger Single Cell Software Suite 10x Genomics Inc (version 8.0.0 and 7.2.0); Reference genome (2020A reference genome, Chromium_Human_Transcriptome_Probe_Set_v1.0.1_GRCh38-2020-A, and refdata-cellranger-vdj-GRCh38-alts-ensembl-7.1.0); cellbender (version 0.2.1); Spaceranger (version 2.1.0); Scanpy (version 1.8.1 and 1.9.3); anndata (version 0.7.6); Scrublet (version 0.2.3); scvi-tools (version 0.20.3); Harmonypy (version 0.0.5); milopy (version 0.1.1); CellTypist (version 1.6.2); CellPhoneDB (version 4.0); cell2location (version 0.7a0); drug2cell (version 0.1.0); infercnv (version 1.6.0); ktplots (version 2.0.0); NMF (version 0.23.0); BayesPrism (version 2.0); edgeR (version 3.32.1); limma (version 3.46.0); Seurat (version 4.0.0); SummarizedExperiment (version 1.20.0); survival (version 3.2-10); survMisc (0.5.5); GraphPad Prism (version 9); survminer (0.4.9)<br><br>Cell sorting decisions were executed by DIVA v.8 (BD Biosciences). FlowJo (v.10.6.2, BD Biosciences) was used for data analysis and presentation. Immunofluorescence microscopy images were analyzed in Inform v.2.4.8 (Akoya) and QuPath v.0.2.3.<br><br>Code Availability:<br>Single-cell sequencing data were processed and analysed using publicly available software packages. The code generated during this study is available at Github: https://github.com/ruoyan-li/Cutaneous-T-cell-lymphoma-study. |

For manuscripts utilizing custom algorithms or software that are central to the research but not yet described in published literature, software must be made available to editors and reviewers. We strongly encourage code deposition in a community repository (e.g. GitHub). See the Nature Portfolio guidelines for submitting code & software for further information.

# Data

Policy information about availability of data

All manuscripts must include a data availability statement. This statement should provide the following information, where applicable:

- Accession codes, unique identifiers, or web links for publicly available datasets
- A description of any restrictions on data availability
- For clinical datasets or third party data, please ensure that the statement adheres to our policy

Data Availability statement
There are no restrictions on data availability for newly generated data presented in this study. FASTQ files of all raw sequencing data from this study have been deposited at EMBL-EBI ArrayExpress and are made publicly available at E-MTAB-12303 and E-MTAB-13614. Previously published scRNA-seq datasets are available in the Genome Sequence Archive for human (accession number HRA000166), NCBI BioProject database (accession number HRA000166) and ArrayExpress (accession number E-MTAB-8142). Previously published bulk RNA-seq datasets are available in the Gene Expression Omnibus database under accession numbers GSE168508 and GSE121212. Scanpy h5ad objects for CTCL, CCL plus skin cell atlas, and Visium data are available for download and can be explored on an online webportal, https://collections.cellatlas.io/ctcl.

# Research involving human participants, their data, or biological material

Policy information about studies with human participants or human data. See also policy information about sex, gender (identity/presentation), and sexual orientation and race, ethnicity and racism.

| | |
|---|---|
| Reporting on sex and gender | Details are described and provided in the methods section 'Patient recruitment and sample acquisition' and Supplementary Table 1. |
| Reporting on race, ethnicity, or other socially relevant groupings | N/A |
| Population characteristics | Details are provided in Supplementary Table 1. |
| Recruitment | Fresh skin samples generated for this study, from patients with CTCL were donated with written consent and approval from the Newcastle and North Tyneside NHS Health Authority Joint Ethics Committee (08/H0906/95+5). Each CTCL patient donated two skin punch biopsies, from a representative plaque or tumour. One biopsy was used for scRNA-seq, and the other for bulk sequencing and IHC. All patients had MF, diagnosed based on correlation of clinical and histopathological features. Stage of CTCL at time of biopsy was taken from the patient's notes and based on clinical assessment performed by dermatology specialists at the Department of Dermatology and NIHR Newcastle Biomedical Research Centre, Newcastle, UK. For fixed sample acquisition for both IHC and single cell flex, FFPE blocks were accessed through the Newcastle CEPA Biobank (17/NE/0070). For additional IHC validation cohorts, samples were donated with consent from the local ethics committee at the Medical University of Vienna (ECS 1360/2018) and the Swedish Ethical Review Authority (2019-03467). CTCL diagnosis and staging as well as monitoring for disease progression was performed by specialists in dermatology and dermato-histopathology at the Department of Dermatology, Medical University of Vienna and the Department of Dermatology, Karolinska University Hospital, Stockholm. There is no recruitment biases in this study. |
| Ethics oversight | Newcastle and North Tyneside NHS Health Authority Joint Ethics Committee and Newcastle CEPA Biobank.<br>local ethics committees at the Medical University of Vienna and the Swedish Ethical Review Authority. |

Note that full information on the approval of the study protocol must also be provided in the manuscript.

# Field-specific reporting

Please select the one below that is the best fit for your research. If you are not sure, read the appropriate sections before making your selection.

☒ Life sciences ☐ Behavioural & social sciences ☐ Ecological, evolutionary & environmental sciences

For a reference copy of the document with all sections, see nature.com/documents/nr-reporting-summary-flat.pdf

# Life sciences study design

All studies must disclose on these points even when the disclosure is negative.

| | |
|---|---|
| Sample size | As per the Human Cell Atlas white paper (https://www.humancellatlas.org/wp-content/uploads/2019/11/HCA_WhitePaper_18Oct2017-copyright.pdf), sample size was determined by recent experience using these technologies in relevant tissues. The sample size was made as large as it could be possible based on the availability of suitable materials. |
| Data exclusions | In this study, data exclusions were limited to removal of low quality single cells for downstream analysis of 'good quality' cells in scRNA-seq datasets. Any such data filtering steps are detailed in full in manuscript 'Methods'.<br>All novel data from this study are publicly available (in both raw and processed forms, as well as on a publicly available on our interactive data portal at https://collections.cellatlas.io/ctcl). |
| Replication | Biological as well as technical replicates were taken which reproduced the same results. We included scRNA-seq data from 45 CTCL patients in the data object as biological replicates. When comparing to healthy skin, AD and psoriasis, we included data from 5 healthy donors, 4 AD patients and 3 psoriasis patients as biological replicates. In IHC validation experiments, 4 to 27 biological replicates were used. |
| Randomization | Randomization was not applicable in this study, because we describe an exploratory analysis in a discovery cohort. |
| Blinding | Blinding for scRNA-seq data generation was not necessary as data were analyzed together using unbiased clustering and annotation of clusters. Analyses with sample characteristics as a variable were not performed until after data were annotated. For other experiments, blinding is not applicable, because we did not perform a clinical study with specific clinical questions. |

# Reporting for specific materials, systems and methods

We require information from authors about some types of materials, experimental systems and methods used in many studies. Here, indicate whether each material, system or method listed is relevant to your study. If you are not sure if a list item applies to your research, read the appropriate section before selecting a response.

## Materials & experimental systems

| n/a | Involved in the study | |
|---|---|---|
| ☐ | ☒ | Antibodies |
| ☒ | ☐ | Eukaryotic cell lines |
| ☒ | ☐ | Palaeontology and archaeology |
| ☒ | ☐ | Animals and other organisms |
| ☒ | ☐ | Clinical data |
| ☒ | ☐ | Dual use research of concern |
| ☒ | ☐ | Plants |

## Methods

| n/a | Involved in the study | |
|---|---|---|
| ☒ | ☐ | ChIP-seq |
| ☐ | ☒ | Flow cytometry |
| ☒ | ☐ | MRI-based neuroimaging |

## Antibodies

| | |
|---|---|
| Antibodies used | BUV395 Mouse Anti-Human CD45, BD Biosciences, Clone HI30, Cat. number 563792<br>Alexa Fluor® 700 anti-human CD8, Biolegend, Clone HIT8a, Cat no. 300920<br>DAPI (nucleic acid stain), Sigma Aldrich, Cat no. D9542-5MG<br>Mouse anti-human CD20 antibody, Clone 26, Dako M0755<br>Mouse anti-human CD79a antibody, Clone JCB117, Dako M7050<br>Rabbit anti-human CD20 antibody, Clone EP459Y, Abcam ab78237<br>Goat secondary anti-Rabbit IgG H&L antibody, Abcam ab214880<br>Rabbit anti-human GTSF1 antibody, Atlas Antibodies, HPA038877<br>Rabbit anti-human TOX antibody, Atlas Antibodies, HPA018322<br>Rarecyte CD31 antibody, ArgoFluor 515, Clone EPR3094, Cat no. 52-1005-501<br>Rarecyte Ki67 antibody, ArgoFluor 555L, Clone D3B5, Cat no. 52-1013-501<br>Rarecyte CD68 antibody, ArgoFluor 535, Clone D4B9C, Cat no. 52-1008-501<br>Rarecyte CD163 antibody, ArgoFluor 580L, Clone EPR14643, Cat no. 52-1009-501<br>Rarecyte CD20 antibody, ArgoFluor 660L, Clone L26, Cat no. 52-1004-601<br>Rarecyte CD4 antibody, ArgoFluor 572, Clone N1UG0, Cat no. 52-1002-501<br>Rarecyte CD8a antibody, ArgoFluor 602, Clone AMC908, Cat no. 52-1003-601 |

Rarecyte CD45RO antibody, ArgoFluor 624, Clone UCHL1, Cat no. 52-1007-601
Rarecyte FOXP3 antibody, ArgoFluor 662, Clone 236A/E7, Cat no. 52-1012-601
Rarecyte CD3e antibody, ArgoFluor 686, Clone D7A6E, Cat no. 52-1001-601
Rarecyte E-Cadherin antibody, ArgoFluor 730, Clone 4A2, Cat no. 52-1010-701
Rarecyte PCNA antibody, ArgoFluor 760, Clone PC10, Cat no. 52-1016-701
Rarecyte CD45 antibody, ArgoFluor 810, Clone D9M8I, Cat no. 52-1006-801
Rarecyte Pan-CK antibody, ArgoFluor 845, Clone C11/AE1/AE3, Cat no. 52-1015-801
Rarecyte Vimentin antibody, ArgoFluor 874, O91D3, Cat no. 52-1019-801
Rarecyte GzmB antibody, ArgoFluor 555L, D6E9W, Cat no. 52-1047-501
Rarecyte CD11c antibody, ArgoFluor 624, D3V1E, Cat no. 52-1046-601
Rarecyte CD138 antibody, ArgoFluor 760, EPR6454, Cat no. 52-1059-701
Rarecyte CD14 antibody, ArgoFluor 810, EPR3653, Cat no. 52-1060-801
(more detailed information for Rarecyte antibodies is listed in Supplementary Table 8)

Validation

BUV395 Mouse Anti-Human CD45: Antibody validated on human peripheral blood lymphocytes for flow cytometry by the manufacturer and previous publications. More information available at https://www.bdbiosciences.com/en-us/products/reagents/flow-cytometry-reagents/research-reagents/single-color-antibodies-ruo/buv395-mouse-anti-human-cd45.563792
Alexa Fluor® 700 anti-human CD8 Antibody: Antibody validated on human peripheral blood lymphocytes for flow cytometry by the manufacturer and previous publications. More information available at: https://www.biolegend.com/en-us/products/alexa-fluor-700-anti-human-cd8a-antibody-3434
Anti-GTSF1 antibody: Antibody validated on human tissues for IHC by the manufacturer and previous publications. More information available at https://www.atlasantibodies.com/products/primary-antibodies/triple-a-polyclonals/anti-gtsf1-antibody-hpa038877-100ul/?language=en
Anti-TOX antibody: Antibody validated on human tissues for IHC by the manufacturer and previous publications. More information available at: https://www.atlasantibodies.com/products/primary-antibodies/triple-a-polyclonals/anti-tox-antibody-hpa018322/
Anti-CD20 antibody: Antibody validated on human B cells for IHC by the manufacturer and previous publications. more information available at: https://www.agilent.com/en/product/immunohistochemistry/antibodies-controls/primary-antibodies/cd20cy-%28concentrate%29-76520
Anti-CD79a antibody: Antibody validated on human B cells for IHC by the manufacturer and previous publications. more information available at: https://www.agilent.com/en/product/immunohistochemistry/antibodies-controls/primary-antibodies/cd79-%28concentrate%29-76625
All antibodies for Rarecyte experiments were validated in-house on human tonsil tissue for IF prior to experiments.

# Plants

Seed stocks

*Report on the source of all seed stocks or other plant material used. If applicable, state the seed stock centre and catalogue number. If plant specimens were collected from the field, describe the collection location, date and sampling procedures.*

Novel plant genotypes

*Describe the methods by which all novel plant genotypes were produced. This includes those generated by transgenic approaches, gene editing, chemical/radiation-based mutagenesis and hybridization. For transgenic lines, describe the transformation method, the number of independent lines analyzed and the generation upon which experiments were performed. For gene-edited lines, describe the editor used, the endogenous sequence targeted for editing, the targeting guide RNA sequence (if applicable) and how the editor was applied.*

Authentication

*Describe any authentication procedures for each seed stock used or novel genotype generated. Describe any experiments used to assess the effect of a mutation and, where applicable, how potential secondary effects (e.g. second site T-DNA insertions, mosiacism, off-target gene editing) were examined.*

# Flow Cytometry

## Plots

Confirm that:

☒ The axis labels state the marker and fluorochrome used (e.g. CD4-FITC).

☒ The axis scales are clearly visible. Include numbers along axes only for bottom left plot of group (a 'group' is an analysis of identical markers).

☒ All plots are contour plots with outliers or pseudocolor plots.

☒ A numerical value for number of cells or percentage (with statistics) is provided.

## Methodology

Sample preparation

Skin biopsies were immediately processed by removing the lower dermis and subcutis and separating epidermis and dermis after dispase II digestion at a concentration of 2U/ml for 2-3 hours at 37oC. Epidermis and dermis were processed separately in type IV collagenase at a concentration of 1.6 mg/ml overnight (37oC 5% CO2). Subsequently, single cell suspensions were formed by vigorous pipetting and filtering (100 micron filter) and counted.
Both cells from the epidermis and dermis were stained with an antibody panel containing CD45 (BD Biosciences) and CD8a (Biolegend) and sorted using FACS into the following fractions: CD45-, CD45+ CD8a+ and CD45+ CD8a-.

Instrument

BD FACSAriaTM Fusion

Software

DIVA v.8 (BD Biosciences)

| | |
|---|---|
| Cell population abundance | Abundance of cell populations were determined post sorting by manually counting. |
| Gating strategy | As shown in the Extended Data Figure 1a, for all FACS experiments, cells were gated based on FSC/SSC, live (DAPI negative set based on unstained cells from the sample) and single cells (FSC-H/FSC-A/SSC-W). For scRNA-seq, cells were taken from both positive and negative gates so to not exclude any cells. |

☒ Tick this box to confirm that a figure exemplifying the gating strategy is provided in the Supplementary Information.
